# Cell death in tumor microenvironment: an insight for exploiting novel therapeutic approaches

**DOI:** 10.1038/s41420-025-02376-1

**Published:** 2025-03-10

**Authors:** Wenxin Wang, Tong Li, Kui Wu

**Affiliations:** 1 BGI Genomics, Shenzhen, 518083 China; 2 Guangdong Provincial Key Laboratory of Human Disease Genomics, Shenzhen Key Laboratory of Genomics, BGI Research, Shenzhen, 518083 China; 3 HIM-BGI Omics Center, Hangzhou Institute of Medicine (HIM), Chinese Academy of Sciences (CAS), BGI Research, Hangzhou, 310030 China

**Keywords:** Cancer therapy, Cell death

## Abstract

Cell death is critical in tumor biology. The common cancer therapies can cause cell death and alleviate tumor, while the cancer cells can develop a resistance to cell death and survive from the therapies. Thus, not only observing the alternative mechanisms of tumor cells resistant to cell death, but also understanding the intricate dynamics of cell death processes within the tumor microenvironment (TME), are essential for tailoring effective therapeutic strategies. High-throughput sequencing technologies have revolutionized cancer research by enabling comprehensive molecular profiling. Recent advances in single cell sequencing have unraveled the heterogeneity of TME components, shedding light on their complex interactions. In this review, we explored the interplay between cell death signaling and the TME, summarised the potential drugs inducing cell death in pre-clinical stage, reviewed some studies applying next-generation sequencing technologies in cancer death research, and discussed the future utilization of updated sequencing platforms in screening novel treatment methods targeted cell death. In conclusion, leveraging multi-omics technologies to dissect cell death signaling in the context of the TME holds great promise for advancing cancer research and therapy development.

## Facts

- Inducing cell death in tumor cells is ideal to kill cell and activate immune response.
- The alteration in TME during cell death is crucial for the treatment effect.
- Potential cells in TME can be good therapeutic targets instead of gene factors in cell death.
- The updated sequencing methods could facilitate the investigations for novel drugs.

## Open questions

- How to investigate therapeutic targets in cell death pathways by advanced sequencing technologies?
- How to eliminate side effects on normal tissues or cells causing by administration of cell death associated treatments?
- How to combine current targeted therapy and immunotherapy methods with potential novel small molecular drugs targeting cell death?

## Introduction

Cell death is essential for maintaining homeostasis during development by eliminating senescent, damaged infected and aberrant cells [1, 2]. The process can be non-immunogenic (apoptosis) and immunogenic and pro-inflammatory (necrosis) [3]. The regulated necrosis is modulated by the genetically programed suicide processes, including necroptosis, proptosis, ferroptosis, cuproptosis and parthanatos [3]. The morphologic, biochemical and molecular features of each cell death fashion vary, and it also can be resulted from a dysregulated metabolism like ferroptosis and cuproptosis. Although the molecular participators of cell death have been investigated for a long time, the cellular behaviors induced by cell death is still underestimated due to a complex multicellular organ. Along with the development of sequencing technology, the researchers can not only investigate gene alterations and modifications associated cell death but also understand cellular behaviors during cell death by single cell sequencing platforms. As given tumor cells often develop a resistance to cell death, the treatments aiming at terminating cell death inhibition are hopeful for enhancing therapy effects as well as combined with targeted-therapy and immunotherapy [4–6]. Besides exploiting therapies targeted on inducing cell death in tumor cells, more candidates in TME can be modulated by regulating cell death and then exert an anti-cancer role. Here we reviewed the discovery and process of major types of cell death, the remodeling effect of cell death in tumor microenvironment and the current therapeutic strategies based on inducing cell death. We tried to provide novel clues to identify certain cell population as therapeutic targets. The flexible combination of traditional therapy, immunotherapy, targeting cell death induction and reshaping the TME may help relieve treatment resistance and obtain better therapy effects. Along with the emerging sequencing technology, the knowledge of both molecular mechanism and TME remodeling associated with cell death keeps being updated. It will be promising to apply updated sequencing technologies in surveying novel treatment targets on both molecular and cellular levels and establish predictive models for patients’ prognosis, therapy responses and precision medicine.

## Cell death in cancers

### Autophagy

Autophagy, a highly conserved cellular process in eukaryotes, enables the sequestration of cytoplasmic components within double-membraned vesicles (autophagosomes) for lysosomal degradation [7]. This process recycles cellular components, including damaged organelles, misfolded proteins, and large macromolecular complexes, to maintain cellular homeostasis. The concept of autophagy was first introduced by Christian de Duve in the early 1960s [8]. He proposed the term “autophagy” after observing double-membrane structures isolating proteolytic enzymes. Subsequent research significantly advanced the understanding of autophagy. In 1993, Yoshinori Ohsumi identified 15 autophagy related genes (ATGs) in yeast through a series of genetic screens [9]. Ohsumi elucidated the functional roles of these genes and their corresponding proteins, laying the groundwork for modern autophagy research. These findings have highlighted autophagy’s evolutionary conservation and its critical roles in physiological and pathological processes, including infection, aging, and various diseases [10].

**Fig. 1:**
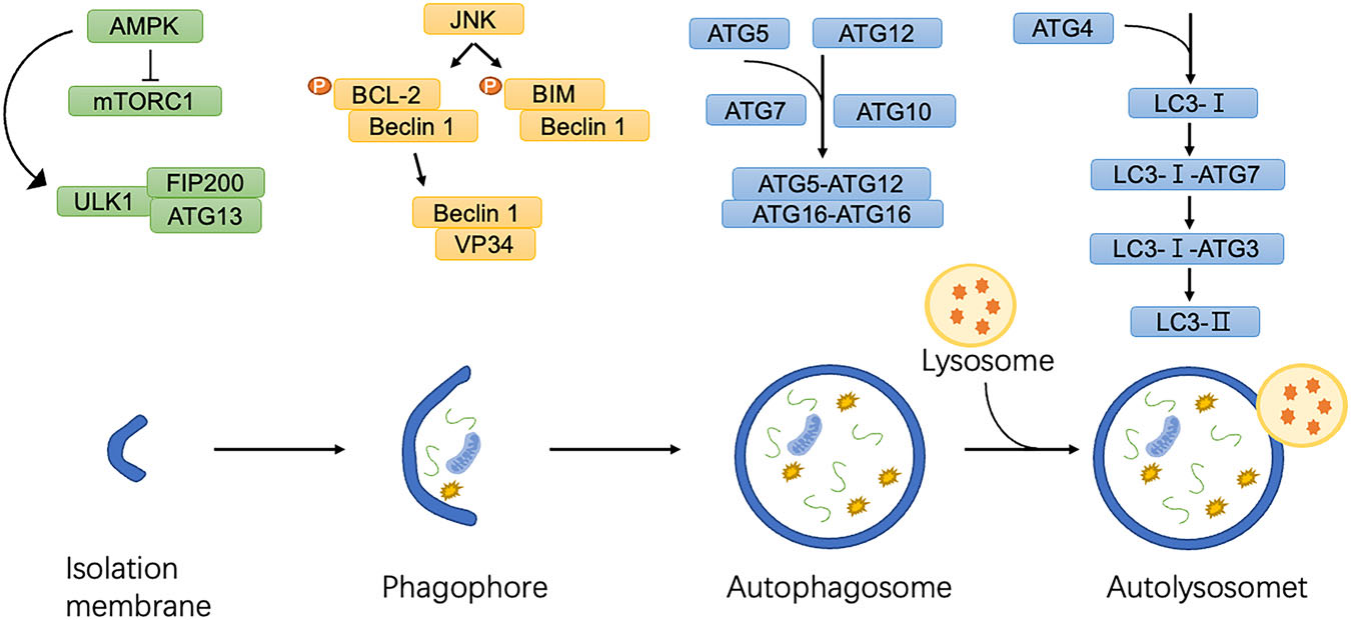
Regulation of autophagy and key proteins involved. Under conditions of nutrient starvation or cellular stress, AMP-activated protein kinase (AMPK) is activated, inhibiting mTORC1 and, in turn, activating the ULK1 complex. The ULK1 complex, composed of ULK1, ATG13, and FIP200, initiates the formation of the phagophore, the precursor to the autophagosome. Concurrently, JNK phosphorylates BCL-2, causing its dissociation from Beclin-1, which activates the PI3K complex and enhances phagophore nucleation. LC3 is conjugated to the phagophore membrane through a lipidation process where LC3-I is converted to LC3-II. This involves conjugation of LC3-I to phosphatidylethanolamine (PE) via the sequential actions of ATG7, ATG3, and the ATG5-ATG12/ATG16L1 complex, enabling autophagosome maturation and cargo recruitment. The mature autophagosome fuses with a lysosome, forming the autolysosome, where lysosomal enzymes degrade the engulfed material, recycling essential macromolecules to maintain cellular homeostasis and energy balance.

Autophagy initiation is tightly regulated by stress signals, including nutrient deprivation, hypoxia, reactive oxygen species (ROS), and DNA damage. The process begins with the activation of the Unc-51-like kinase (ULK) complex, comprising ULK1/ULK2, ATG13, ATG101, and FIP200 [11] (Fig. 1). This complex recruits and activates the vacuolar protein sorting 34 (VPS34) complex, which includes key subunits such as Beclin-1 (BECN1) and PIK3R4, facilitating the synthesis of phosphatidylinositol-3-phosphate (PI3P) at nascent autophagic membranes [12]. Autophagosome elongation and maturation involve two ubiquitin-like conjugation systems. The first system mediates the attachment of ATG12 to ATG5, forming an ATG12–ATG5 complex, which binds to ATG16 to anchor to the autophagosomal membrane [13]. The second system involves the light chain 3 (LC3) and GABARAP families [14]. LC3 is conjugated to phosphatidylethanolamine (PE), driving autophagosome expansion and cargo sequestration. Once mature, autophagosomes fuse with lysosomes to form autolysosomes, where lysosomal hydrolases degrade the sequestered material, releasing basic components back into the cytoplasm for reuse [7].

While autophagy primarily functions as a protective mechanism, excessive or dysregulated autophagy can lead to cell death. Autophagy-dependent cell death (ADCD) is a form of regulated cell death (RCD) that relies on autophagic machinery [15]. The Nomenclature Committee on Cell Death in 2018 defined ADCD as a process mechanistically dependent on autophagy components [16]. Despite its capacity to induce cell death, autophagy is not inherently a death mechanism. Instead, it serves as a critical cellular defense mechanism against various stresses. Dysregulation of autophagy can result in pathological outcomes, including cancer, where impaired autophagy may enable tumorigenesis by disrupting cellular homeostasis [17]. Conversely, excessive autophagy can lead to degradation of essential cellular components, causing irreversible damage and cell death.

Autophagy is a vital cellular process for maintaining energy and metabolic homeostasis by recycling intracellular components. While it primarily serves protective roles, autophagy can induce cell death under certain conditions, particularly through ADCD mechanisms [18]. However, it is crucial to distinguish these forms of autophagy-related cell death from other RCD modalities.

### Apoptosis

In 1972, Kerr, Wylie and Currie first defined an initiative cell death as apoptosis, which caused classical morphological alterations during cells dying under physiological conditions, including condensed nuclear and cytoplasma, and broken cells with the formation of a number of ultrastructural well-preserved fragments with membrane [19]. Subsequently, ced-3 and ced-4 were identified as initiators of apoptosis in nematodes *C. elegans*, which were correspondent to caspase-3 and caspase-9 genes in mammals [20, 21]. It was a breakthrough to discover the caspase family of cysteine proteases which exert critical roles in apoptotic signaling and execution [22].

**Fig. 2:**
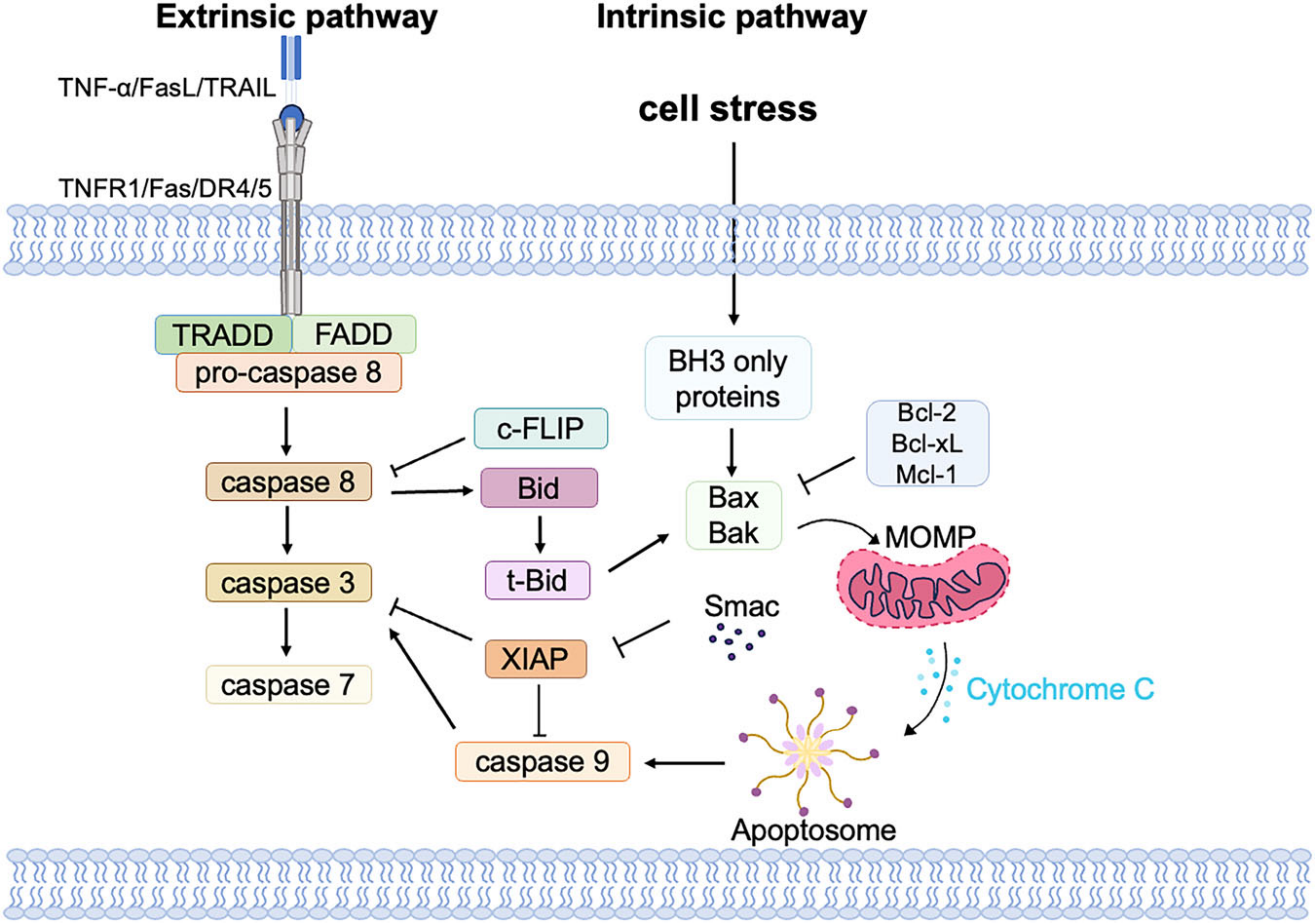
Molecular mechanisms of apoptosis pathway activation. The extrinsic pathway is the binding of various cytokines including TNF-α, TRAIL and CD95 to their respective receptors, and the death receptors have a death domain for recruiting downstream adaptor proteins (FADD,TRADD) and pro-caspase 8 to form a death inducing signaling complex, which leads to the activation of caspase-8 and further activates caspase3 and caspase 7.The intrinsic pathway is activated by various stresses and induces transcription and translation of pro-apoptotic members of the BCL-2 family, which binds to Bcl-2, Bcl-XL, Mcl-1, and release BAK and BAX, which then assemble to form complexes that lead to MOMP and the release of apoptotic factors cytochrome c and Smac. Smac is able to relieve the restriction of caspase by XIAP, and cytochrome c binds to the cell membrane protein APAF1 to form an apoptotic complex (apoptosome) and activate caspase 9.

Two major apoptotic pathways have been described: the extrinsic and intrinsic ones [23, 24] (Fig. 2). The intrinsic pathway is also known as mitochondrial pathway and regulated by B-cell lymphoma-2 (BCL-2) family proteins BAK and BAX [25]. When cells are exposed to intracellular stimuli, such as toxic agents or DNA damage, BH3-only proteins are activated. The proteins promote the homodimerization and oligomerization of BAX and BAK at the outer mitochondrial membrane, a process termed mitochondrial outer membrane permeabilization (MOMP) [6, 26]. MOMP then stimulates the release of cytochrome c from mitochondria [23]. Cytochrome c with apoptotic protease-activating factor-1 (APAF1), procaspase-9 and dATP forms the apoptosome complex together [20]. Subsequently, monomeric procaspase-9 is dimerized by the apoptosome and then processes autocatalytic cleavage to form a heterotetrameric complex. The activated caspase-9 cleaves and activates caspase-3 in turn, resulting in the morphologic alterations of apoptotic cell death [20]. Moreover, the second mitochondria-derived activator of caspase (SMAC), released with cytochrome c during MOMP, functions as an inhibitor of apoptosis protein-binding protein to promote apoptosis [27].

The extrinsic pathway, also named the death receptor pathway, primarily involves death signals such as FAS ligand (FasL), tumor necrosis factor α (TNF-α) and TNF-related apoptosis-inducing ligand (TRAIL) [28–30]. These factors bind to death receptors (DRs), including TNFR1/2, FAS, and TRAIL receptors DR4 and DR5 located on the cell membrane [31–34]. This leads to the adapter proteins gathering and activating the apoptotic initiator caspases, caspase-8 and caspase-10, forming the death-inducing signaling complex (DISC) [35–37]. Activation of caspase-8 and caspase-10 triggers downstream effector caspases, including caspases-3 and caspase-7 [38]. The effector caspases trigger a series of intracellular events, including DNA fragmentation and changes in the cell membrane, ultimately leading to cell death. The extrinsic apoptotic pathway can intersect with the intrinsic apoptotic pathway through caspase-8-mediated proteolytic activation of the pro-apoptotic BH3-only protein BID [30].

As a crucial process in cell renewal, embryonic development and immune system functioning, apoptosis also acts as an essential step in cancer therapy-induced cell death [39]. Unfortunately, in some cases, tumor cells can develop resistance to apoptosis and escape from the immune surveillance and drug toxicity. The tumor microenvironment plays a significant role in the process, affecting the susceptibility of tumor cells to apoptosis induced by chemotherapy. Although apoptosis is essential for cancer therapy, limited apoptosis within the tumor cell population can have opposing effects to promote cell survival and therapy resistance by shaping the TME. This influence extends to phagocytes, viable tumor cells, and the stimulation of pro-oncogenic effects. Notably, the continuous activation of innate immune response by apoptosis may shape a pro-oncogenic TME and boost the evasion of drug treatment. Apoptosis and caspase activation correlate with aggressive disease in multiple malignancies [39].

### Necroptosis

Necroptosis, a type of programmed necrosis, is known to be more pro-inflammatory than apoptosis [40, 41]. This process is characterized by morphological changes, such as increased cell volume, organelle swelling, and rupturing of the plasma membrane [41]. Necrosis is considered as a passive, unregulated way of cell death, but further investigation revealed that a cell death mechanism resembling necrosis can be subject to regulation through a specific intracellular program [42]. The scientists observed programmed necrosis for a long time, while a compound called Necrostatin-1 (Nec-1) was discovered that could inhibit TNF and z-VAD-induced programmed necrosis in a variety of cells until 2005. They subsequently coined the term “necroptosis” to describe this caspase-independent programmed necrosis [43]. In 2009, three independent teams reported that receptor-interacting serine-threonine kinase 3 (RIP3) acts as a downstream substrate of RIP1 to mediate the transmission of the necroptosis signaling pathway [44–46]. Mixed lineage kinase domain-like pseudokinase (MLKL) was identified as a key downstream component of TNF-induced necroptosis [47, 48].

**Fig. 3:**
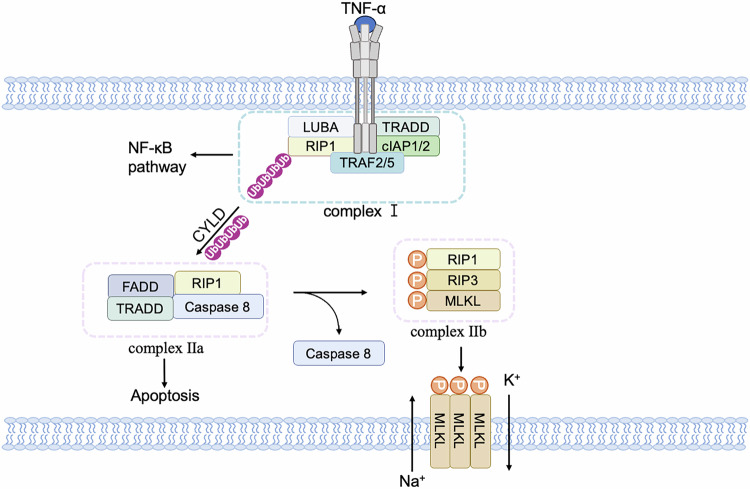
TNF-induced necroptosis signaling pathway. After TNFα binds to TNFR1, it forms a TNFR1 signaling complex by recruiting TRADD, RIP1, and TRAF2/5, activating downstream signaling pathways such as NF-$\kappa$B. The complexes of TRADD, RIP1, and TRAF2 proteins dissociate from receptors and recruit other proteins to compose distinct secondary complexes to regulate apoptosis and necroptosis. Among them, apoptosis is mainly initiated by the recruitment of FADD, which recruits and activates caspase 8 to cause apoptosis, while necroptosis requires the activation of RIP3 and the recruitment of downstream MLKL proteins. Phosphorylated MLKL proteins migrate to the cell membrane, causing K^+^ efflux and Na^+^ influx.

Necroptosis can be induced by different innate immune signaling pathways, including RIG-I-like receptors, toll-like receptors (TLRs), and death receptors [49]. All these pathways lead to the phosphorylation and activation of RIP3 and trigger the necroptosis signaling pathway [50]. In the case of necroptosis induced by death receptors, the stimulation of TNF signaling results in the recruitment of TNFR1-associated death domain (TRADD), cellular inhibitor of apoptosis protein 1 (cIAP1), cIAP2, TNF-receptor-associated factor 2 (TRAF2), and TRAF5 to form complex I [51] (Fig. 3). When RIP1 is deubiquitinated by the deubiquitinase cylindromatosis (CYLD), the NF-$\kappa$B pathway is constrained, leading to the formation of complex II consisting of RIP1, TRADD, caspase 8, and FAS-associated death domain protein (FADD) [50, 52]. RIP1 self-phosphorylation then activates RIP3, forming necrosomes, and further activates downstream MLKL, causing a conformational change that transports it to the plasma membrane and induces membrane permeabilization [53–57]. As a result, sodium ions (Na^+^) influx and potassium ions (K^+^) efflux disrupt the cell membrane potential and ultimately lead to cell rupture [58, 59]. The release of cellular contents can trigger inflammation and activate immune responses [60].

Necroptosis plays a complex role in the TME. This process induces the death of tumor cells, ultimately inhibiting tumor growth and metastasis. Subsequently, the tumor cells undergoing necroptosis are recognized and phagocytosed by phagocytes, dendritic cells, macrophages, monocytes, and neutrophils, leading to releasing pro-inflammatory cytokines and chemokines, as well as stimulatory factors, improving cross-presentation, and ultimately activating adaptive immune responses [61, 62]. Yatim et al. explored the role of RIP1 and NF-$\kappa$B signaling in the process of cross-priming CD8^+^ T cells [63]. They utilized various knockout mouse models to evaluate the impact of these signaling pathways on CD8^+^ T cell responses. They observed that RIP1-deficient dying cells exhibited impaired cross-presentation and reduced activation of CD8^+^ T cells by antigen presenting cells (APCs). Furthermore, they found that RIP1-mediated NF-$\kappa$B signaling in dying cells was critical for the upregulation of genes involved in antigen presentation and T cell activation. Similarly, Aaes et al. investigated the potential of using necroptotic cancer cells as a vaccination strategy to stimulate effective anti-tumor immune responses [64]. They found that necroptotic cancer cells released various danger signals and pro-inflammatory molecules, such as high-mobility group box 1 (HMGB1) and ATP, which promoted the activation of dendritic cells and the recruitment of immune cells to the tumor site. The vaccinated mice exhibited an increased frequency of tumor-specific cytotoxic T cells, indicating a successful priming of the immune system against tumor antigens. Importantly, these cytotoxic T cells demonstrated potent tumor-killing activity, leading to significant suppression of tumor growth and improved survival rates in vaccinated mice.

However, the induction of necroptosis also generates an immunosuppressive tumor microenvironment that promotes tumor growth. Seifert, L. et al. investigated how the necrosome influences pancreatic oncogenesis, shedding light on potential targets for intervention [65]. They utilized genetically engineered mouse models and human pancreatic cancer cell lines to study the role of specific necrosome components, including RIP3 and MLKL, in pancreatic tumor growth and immune evasion. The study revealed that the necrosome promotes pancreatic oncogenesis through two distinct mechanisms. Firstly, the necrosome activation in pancreatic cancer cells led to the secretion of CXCL1, a chemokine that promotes tumor growth and metastasis. CXCL1 enhanced the recruitment of myeloid-derived suppressor cells (MDSCs), a population of immune cells that play a suppressive role in anti-tumor immune responses, thereby creating an immunosuppressive microenvironment conducive to cancer progression. Secondly, the necrosome triggered the expression of Mincle, a C-type lectin receptor, which facilitated immune evasion by inhibiting the activation of dendritic cells and impairing their ability to initiate T cell responses against the tumor. The findings underscore the importance of understanding the intricate interplay between necroptosis death mechanisms, tumor microenvironment, and immune regulation in cancer progression. Further exploration of the necrosome pathway and its downstream effects may pave the way for the development of novel therapeutic interventions for malignant tumors with dysregulated necroptosis.

### Pyroptosis

Pyroptosis is a type of programmed cell death that plays an important role in innate immunity and inflammation. The process was initially misinterpreted as apoptosis when the scientists observed that the invasive bacterial pathogen *Shigella flexneri* can induce host cell death [66, 67]. Brennan and Cookson provided evidence that *Salmonella typhimurium-*induced cell death in macrophages was distinct from apoptosis, causing a diffuse pattern of DNA fragmentation and rupture of cell membranes, and that this death process was dependent on caspase-1 [68]. Then they proposed the term “pyroptosis” from the Greek roots “pyro” and “ptosis” to describe the pro-inflammatory programmed cell death [69]. Over the next decade, more efforts were pay to illustrate the molecular mechanism of pyroptosis [70, 71].

**Fig. 4:**
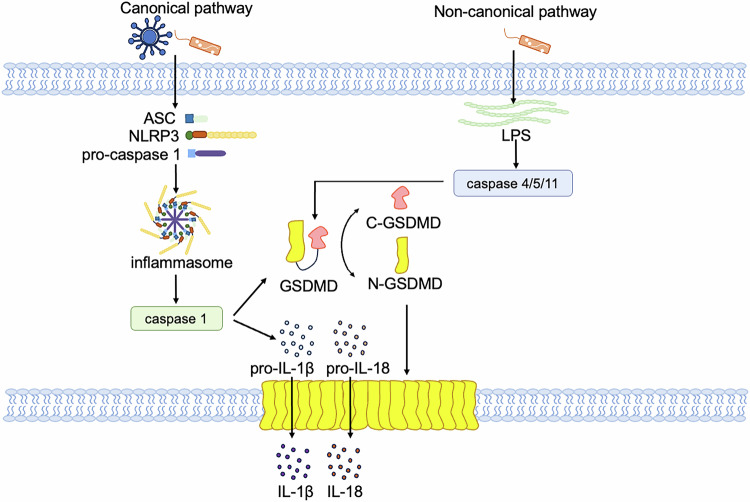
Molecular mechanisms of canonical and non-canonical pathways in pyroptosis. In canonical pathway, when a pathogen invades the host cell, ASC, NLRP3 and caspase 1 recruitment are triggered to form inflammasome, in which caspase 1 is activated. Activated caspase 1 directly cleaves GSDMD and the precursor cytokines pro-IL-1β and pro-IL-18, induces pyroptosis, and promotes the maturation of IL-1β and IL-18. Lysed GSDMD-NTs form pores in the cell membrane that mediate the release of cytoplasmic contents. The non-canonical pathway refers to caspase 4 or caspase 5 in human cells or caspase 11 in mouse cells that recognize LPS, and these inflammatory caspases directly cleave GSDMD and trigger pyroptosis.

Pyroptosis is regulated through either the canonical or non-canonical pathway (Fig. 4). The canonical pathway is facilitated by the assembly of the inflammasome, resulting in the cleavage of gasdermin D (GSDMD) and the subsequent release of IL-1β and IL-18 [72]. Pathogen-associated molecular patterns (PAMPs) and damage-associated molecular patterns (DAMPs) receive intracellular signaling molecule stimulation and lead to assembly with pro-caspase-1 and the adaptor molecule apoptosis-associated speck-like protein (ASC), resulting in the formation of inflammasomes and the activation of caspase-1 [73–77]. Cleaved caspase-1 subsequently targets pro-IL-1β/IL-18 and GSDMD, which is a pore-forming protein that is normally kept inactive by the N-terminal domain. The N-terminal fragment of GSDMD (N-GSDMD) forms nonselective pores, perforating the cell membrane and inducing water influx, lysis, and cell death. Moreover, IL-1β and IL-18 are secreted through the pores created by N-GSDMD [71, 78]. In non-canonical pathway, pyroptosis is initiated by the activation of inflammatory caspases, primarily caspase-11 (in mice) or caspase-4 and caspase-5 (in human), which are activated by various stimuli such as bacterial toxins, viruses, or cytosolic DNA [79]. Upon activation, these caspases cleave GSDMD. Cleavage of GSDMD by these caspases generates a 31 kDa N-terminal fragment which initiates pyroptosis, and a 22 kDa C-terminal fragment, resulting in the formation of large pores in the plasma membrane, leading to osmotic lysis and release of inflammatory contents [71, 80, 81]. However, caspase-4/5/11 cannot cleave pro-IL-1β/pro-IL-18 directly, they mediate the maturation and secretion of IL-1β/ IL-18 through the NLRP3/caspase-1 pathway [82–84].

Pyroptosis is associated with multiple cancers, including breast cancer, colorectal cancer, and gastric cancer [85]. GSDMB, GSDMC, and GSDME have been reported as potential prognostic markers for breast cancer [86–88]. Antibiotics such as doxorubicin could upregulate the expression of PD-L1 and GSDMC, and activate caspase-8, and then cause a pyroptotic death in breast cancer cells. The long-term infection of *Helicobacter pylori* can promote the progression of multiple gastric and extra-gastric diseases through the activation of the NLRP3 inflammasome and the release of cytokines such as IL-1β, IL-6 [89–91].

The effects of pyroptosis on tumors, whether promoting or suppressing, primarily depend on various factors including the tumor type, host inflammatory status, immune response, and the specific effector molecules involved. Wang et al. utilized the Phe-BF3 desilylation bioorthogonal system, enabling specific labeling of pyroptotic cells in live organisms, to investigate the impact of pyroptosis on antitumor immune responses [92]. The authors verified that this system released gasdermin from NP–GSDMA3 to induce pyroptosis and performed single-cell sequencing to reveal changes in immune cell subsets and gene expression after the occurrence of pyroptosis. The numbers of CD4^+^, CD8^+^, and natural killer cells were increased, while monocytes, neutrophils, and MDSCs were decreased. The expression of lymphocyte activation-related pro-inflammatory factors were upregulated, while tumor-promoting genes and immunosuppressive genes were downregulated. Zhang et al. proved that pyroptosis is immunogenic cell death (ICD) and that the expression of GSDME in tumors can inhibit tumor growth and promote the activity of CD8^+^ T cells and NK cells [93]. After pyroptosis, the cell not only releases ATP as a “find me” signal to attract macrophages to the tumor site but also induces phosphatidylserine externalization as an “eat me” signal for phagocytosis of tumor cells [94]. In addition to serving as a “find me” signal to attract macrophages, ATP can also bind to P2RX7 receptors on the surface of dendritic cells, leading to the release of IL-1β and stimulation of CD8^+^ T cells to release IFN-γ, exerting antitumor effects [95]. Understanding the complex interplay of pyroptosis within the TME carries substantial implications for customizing innovative therapeutic approaches. Focusing on pyroptotic pathways or leveraging the immunogenic capacity of pyroptotic cells presents a hopeful avenue for propelling the field of cancer immunotherapy forward.

### Ferroptosis

Ferroptosis is a regulated form of cell death that relies on iron and is triggered by the detrimental build-up of reactive oxygen species (ROS) derived from lipids [96]. This process occurs when the repair systems for lipid peroxides, which are dependent on glutathione (GSH), become compromised [96].

Between 2001 and 2008, the Stockwell Lab screened two new compounds and named them “erastin” and “RAS synthetic lethal 3 (RSL3)” which could induce a regulated, but non-apoptotic, iron-dependent cell death [97–99]. Morphologically, ferroptosis is characterized by significant mitochondrial shrinkage, increased membrane density, and the loss or reduction of mitochondrial cristae. Subsequently the term “ferroptosis” was introduced in 2012 to describe this mode of cell death characterized by the accumulation of ROS and triggered by erastin and RSL3 [100]. This discovery led to the development of the initial small molecule ferroptosis inhibitor known as ferrostatin-1 [100].

**Fig. 5:**
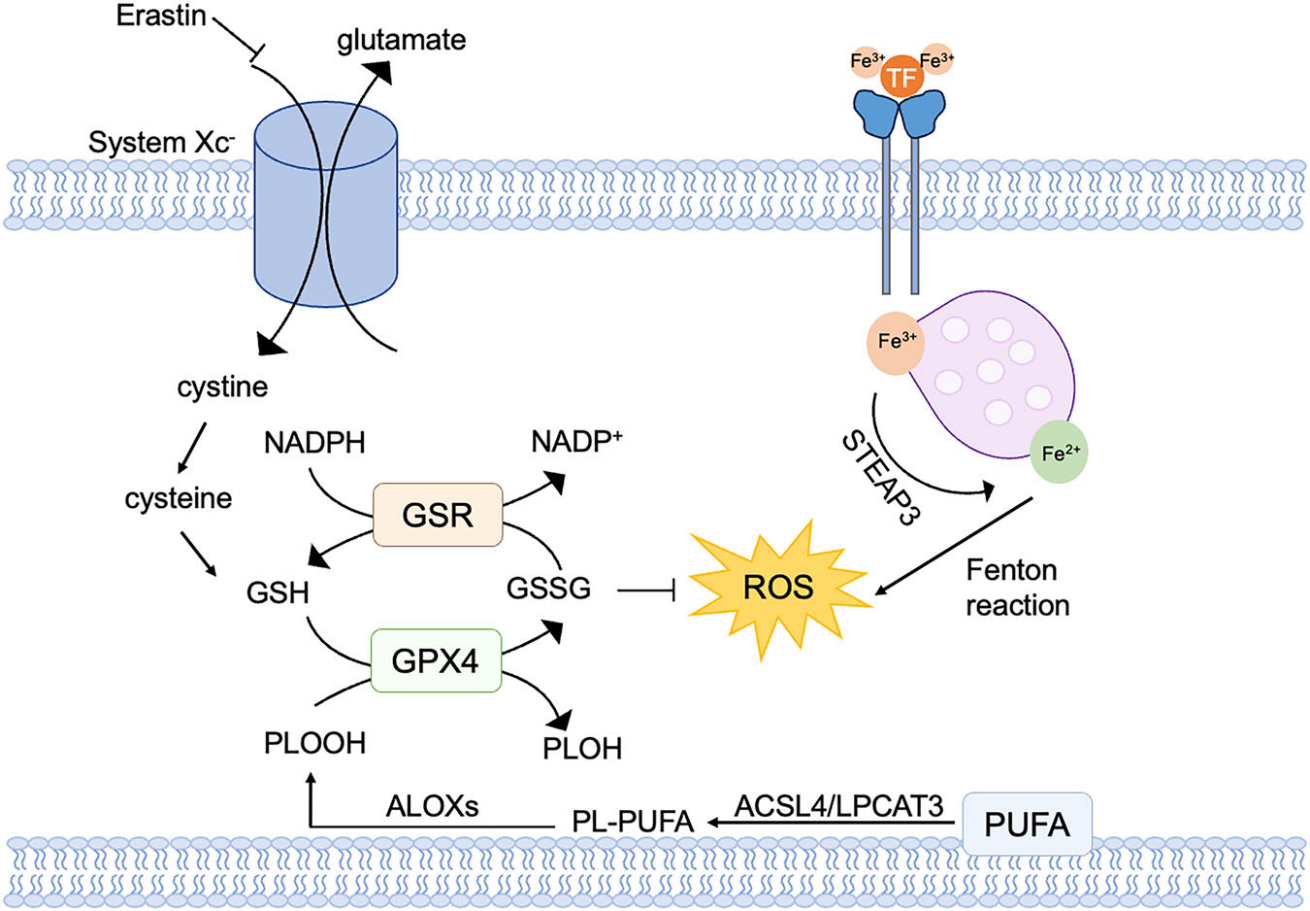
Molecular mechanisms and regulatory networks of ferroptosis. Cystine enters cells via system Xc^-^, where it is reduced to GSH by thioredoxin reductase 1 (TXNRD1)-dependent cystine-reducing pathway. GSH is a potent reducing agent that acts as a cofactor for GPX4, promoting the intracellular reduction of phospholipid hydroperoxides (PLOOHs) to the corresponding alcohols (PLOHs) of PLOOHs. Glutathione-disulfide reductase (GSR) utilizes electron-catalyzed oxidized glutathione (GSSG) provided by NADPH to regenerate GSH. Overloading of iron transporters causes an increase in intracellular iron ions, which induces the Fenton reaction, where the resulting peroxy radicals attack lipid molecules and oxidize them to lipid peroxides.

The induction of ferroptotic cell death involves targeting two cellular components, namely system Xc- and GPX4, which can be inhibited by erastin and RSL3. System Xc- is a widely distributed amino acid antitransporter found in phospholipid bilayers. It plays a crucial role in the cellular antioxidant system. To restrain the activity of system Xc- can disrupt the synthesis of glutathione by blocking the absorption of cystine. This inhibition results in decreased activity of glutathione peroxidase (GPX), reduced cell antioxidant capacity, accumulated lipid ROS, and finally oxidative damage and ferroptosis [101, 102]. GPX4, among the numerous members of the GPX family, plays a crucial role in the initiation of ferroptosis and serves as the primary regulator. Inhibition of GPX4 activity results in the accumulation of lipid peroxides, which serves as a hallmark of ferroptosis (Fig. 5). GPX4 converts GSH into oxidized glutathione (GSSG) and reduces cytotoxic lipid peroxides (PL-OOH) into the corresponding alcohols (PL-OH) [103]. RSL3, compounds DPI7 and DPI 10 can inhibit GPX4 activity and induce ferroptosis [104]. The mevalonate pathway regulates the maturation of selenocysteine tRNA which is participated in the synthesis of GPX4. The inhibition of mevalonate pathway can also downregulate GPX4 and trigger ferroptosis. The erastin can affect voltage-dependent anion channels (VDACs) to induce an unbalance of ions and mitochondrial dysfunction. The oxides would be accumulated in cells eventually resulting in ferroptosis [104]. Recently, it has been discovered that acetylation-deficient mutants of p53 promote ferroptosis. P53 can inhibit the uptake of cystine through system Xc- by downregulating the expression of SLC7A11, thereby affecting the activity of GPX4. This downregulation leads to a reduction in antioxidant capacity, an accumulation of ROS, and then ferroptosis [102]. Additionally, the p53-SAT1-ALOX15 pathway is also involved in the regulation of ferroptosis [105]. Fe^3+^ binds to transferrin (TF) in the serum and then is recognized by the transferrin receptor (TFRC) in the cell membrane. Intracellularly, the STEAP3 metalloreductase in the endosome reduces Fe^3+^ to Fe^2+^, and then Fe^2+^ is released into the cytoplasma through solute carrier family 11 member 2 (SLC11A2/DMT1). Abnormal accumulation in Fe2^+^ in lysosomes and endoplasmic reticulum (ER) may trigger ferroptosis. The polyunsaturated fatty acid (PUFA) is catalyzed by long-chain fatty acid–CoA ligase 4 (ACSL4) and lysophospholipid acyltransferase 5 (LPCAT3), to form phospholipids-polyunsaturated fatty acid (PL-PUFA) [106, 107]. Subsequently, PL-PUFA is oxygenated by arachidonate lipoxygenases to produce phospholipid hydroperoxides (PL-PUFA-OOH), which can promote ferroptosis [108].

Several studies have shown that ferroptosis can enhance antitumor immunity [109–111]. Wang et al. identified that effector CD8^+^ T cells could promote tumor cell ferroptosis through the generation of interferon-gamma (IFNγ), which inhibited the glutamate–cystine antiporter system Xc^-^ and led to lipid peroxidation and ferroptosis [112]. They demonstrated that immunotherapy-activated CD8^+^ T cells played a central role in promoting ferroptosis-specific lipid peroxidation, thereby enhancing the efficacy of immunotherapy against tumors. They revealed that the co-culture of activated CD8^+^ T cells with tumor cells led to an increase in lipid ROS and subsequent induction of tumor cell ferroptosis. Activated CD8^+^ T cells can secrete IFNγ and TNF. Using anti-IFNγ antibodies, anti-TNF antibodies, and CRISPR technology, the researchers confirmed IFNγ as the primary mediator of tumor cell ferroptosis. In addition, IFNγ, by upregulating interferon-regulatory factor 1 (IRF1) and downregulating the expression of SLC7A11 and SLC3A2, modulated the function of system Xc-, leading to tumor cell ferroptosis via the Janus kinase (JAK) and signal transducer and activator of transcription 1 (STAT1) signaling pathway within tumor cells. The research highlights the critical role of CD8^+^ T cell in tumor ferroptosis promotion as an anti-tumor mechanism. Understanding the interplay between CD8^+^ T cells and ferroptosis in the context of cancer immunotherapy holds great promise for enhancing therapeutic outcomes.

During the immune checkpoint inhibitors (ICB) treatment of cancer, the poor efficacy is often due to the dysregulation of CD8^+^ T cells in the immune microenvironment [113, 114]. Inhibitory ligand expression, metabolic factors, and suppressive compounds in the immune microenvironment limit the immune response and affect the function of tumor-infiltrating T cells [115]. One intriguing aspect related to ferroptosis is the role of CD8^+^ T cells in lipid metabolism. CD36, a versatile cell-surface receptor known for its multifunctional roles, plays a pivotal role in the high-affinity uptake of long-chain fatty acids in tissues, contributing to lipid accumulation and metabolic disturbances [116, 117]. Recent studies have shown that tumor infiltrating CD8^+^ T cells exhibited an upregulation of CD36 expression compared with T cells in normal tissues from the same patient and the cholesterol in the TME contributed to upregulating CD36 expression on CD8^+^ T cells [116, 117]. In mouse model, CD36^−^ CD8^+^ T cells have stronger antitumor function than CD36^+^ CD8^+^ T cells in vivo and have a lower expression level of genes associated with the activation of lipid peroxidation and ferroptosis. These findings suggest that the interplay between CD8^+^ T cells, lipid metabolism, and ferroptosis within the TME may hold the key to enhance cancer immunotherapy outcomes, shedding new light on potential strategies to harness the power of ferroptosis for targeted cancer treatment.

### Cuproptosis

Copper is an essential metal factor in cell biology [118]. The overload copper ions in cells can induce regulated cell death, such as apoptosis, paraptosis, pyroptosis, ferroptosis, and cuproptosis [119]. It was initially thought that copper ionophores, such as disulfiram and elesclomol, induced cell death by affecting mitochondria to produce ROS, but the specific mechanism remained unclear [120]. In 2019, Tsvetkov et.al. explored which elesclomol exerted its antitumor effects in a myeloma mouse model to discover the mechanisms. Elesclomol is known to transport copper ions into cells, and its interaction with the mitochondrial enzyme ferredoxin 1 (FDX1) plays a central role in copper’s cytotoxic effects. Elesclomol-bound copper Cu²⁺ is reduced to Cu⁺ by FDX1, which then accumulates in the mitochondria [121]. The term “cuproptosis” was coined in 2022 by Tsvetkov and colleagues to describe this novel form of cell death induced by copper overload [122].

**Fig. 6:**
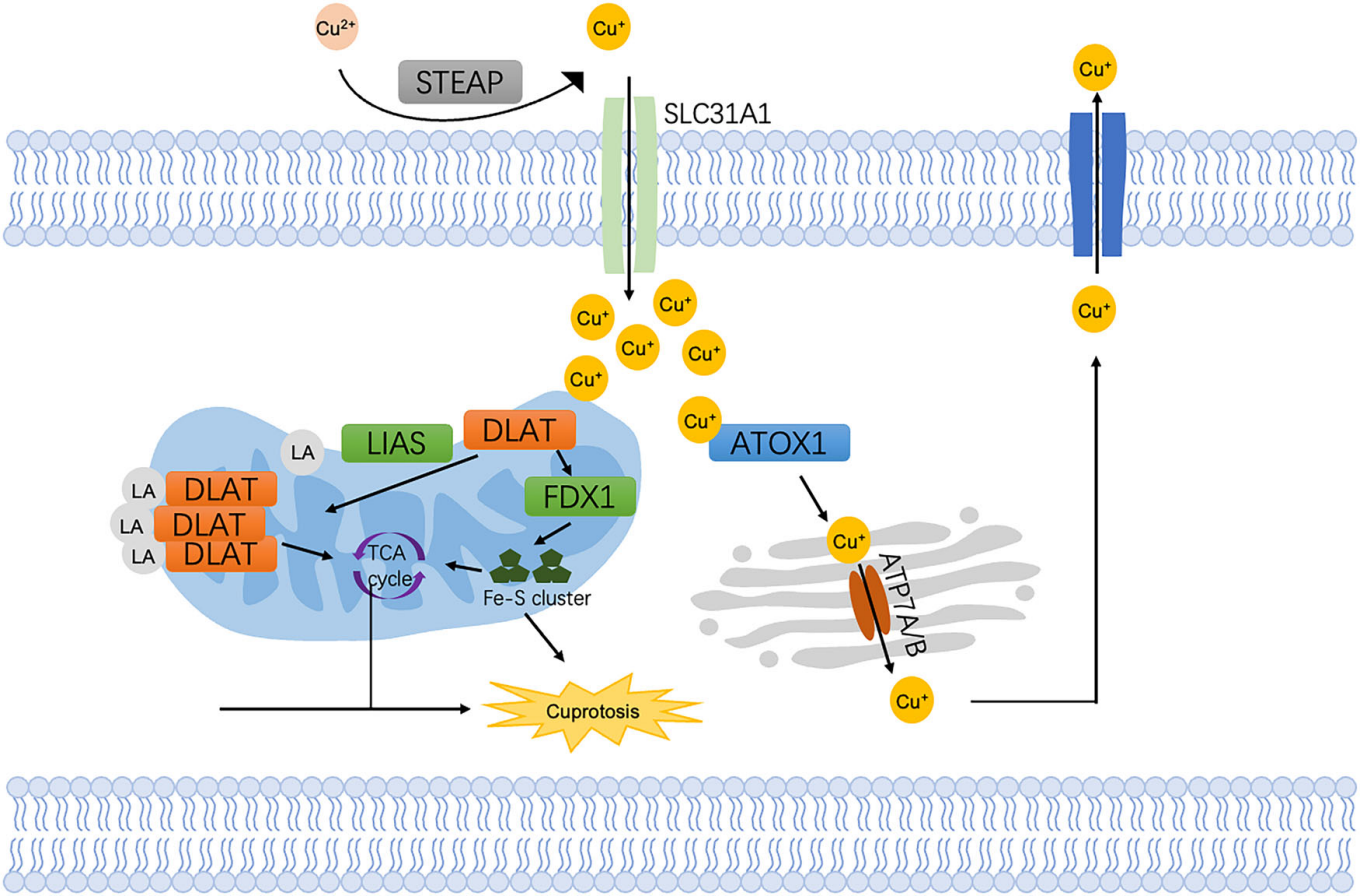
A simplified schematic representation of the molecular mechanism underlying cuproptosis. The reduced Cu⁺ is then transported into the cell by the copper transporter SLC31A1 (CTR1). Inside the mitochondria, Cu⁺ accumulates and binds to dihydrolipoamide S-acetyltransferase (DLAT), leading to its aggregation. This disrupts mitochondrial function, including the synthesis of iron-sulfur clusters (Fe-S) and leads to the generation of excessive ROS. Excessive lipoylated DLAT leads to the accumulation of toxic protein aggregates in mitochondria, inducing cuproptosis. In the Golgi apparatus, ATP7A/B transports Cu^+^ into the lumen for incorporation into cuproenzymes or to be exported out of the cell.

Cuproptosis is distinct from other well-established cell death mechanisms such as apoptosis, necroptosis, ferroptosis and pyroptosis (Fig. 6). The first step in cuproptosis involves the entry of copper ions into the cell, which is crucial for various cellular processes as well. Copper ions can enter cells through copper transporters, such as CTR1 (copper transporter 1), or via copper ionophores, which act as carriers to facilitate copper ion entry into cells. Once entering the cell, copper ions are typically transported to the mitochondria, where they accumulate and interact with FDX1. FDX1 is a key protein involved in the electron transport chain, and it plays a critical role in reducing copper ions (Cu²⁺) to their more reactive form, copper(I) (Cu⁺). The hallmark of cuproptosis is the interaction of copper(I) with lipoylated proteins in the tricarboxylic acid cycle (TCA cycle). One of the main proteins that copper(I) interacts with is dihydrolipoamide S-acetyltransferase (DLAT), a component of the pyruvate dehydrogenase complex (PDC) in the TCA cycle. Another critical aspect of cuproptosis is the destabilization of iron-sulfur (Fe-S) clusters. Many enzymes involved in the TCA cycle and other mitochondrial processes rely on these Fe-S clusters for their activity. When copper(I) interacts with the lipoylated TCA cycle proteins, it destabilizes the Fe-S clusters, leading to proteotoxic stress. The cell is overwhelmed by the accumulation of dysfunctional proteins that cannot be properly degraded or refolded, triggering a cascade of molecular events leading to cell death [122].

To sustain their rapid proliferation, tumor cells regulate copper levels by increasing copper uptake, altering its distribution, and enhancing its metabolism [118]. Studies have demonstrated that serum copper ion levels are significantly elevated in patients with lung cancer, prostate cancer, breast cancer, gallbladder cancer, gastric cancer, and thyroid cancer [123–128].

## Targeting cell death in cancer therapy

### Autophagy

The effect of autophagy in tumors is a double-edged sword. In the initial stage of tumor progression, the occurrence of autophagy can help restrain tumor cell growth, while it can induce drug resistance to facilitate tumor cell survival in the late stage [129]. As given ULK1 is a key factor of autophagy cascade, the inhibitors of ULK1 have showed potential effect in controlling tumor. For instance, SBP-7455 was proved to inhibit the metastatic breast cancer cell growth through alleviating ULK1-mediated protective autophagy [130]. Some natural compounds showed therapeutic potency as participated in blocking autophagy and overcoming drug resistance in cancer cells. Phloretin could restrain cytotoxic autophagy by inhibiting the mTOR/ULK1 pathway, which improved the susceptibility of breast cancer cells to chemotherapies [130].

To induce autophagy can also serve as an effective method to cause cell death and suppress tumor growth. ABTL0812 is an inhibitor of mTORC pathway which was found to improve autophagy-dependent cell death in lung cancer and pancreatic cancer cells [131]. Ginsenoside was found to enhance the LC3-dependent autophagy by blocking PI3K/Akt/mTORC1 pathway and induce apoptotic cell death in osteosarcoma cells [130]. W922 can also inhibit colorectal cancer cell proliferation by inducing autophagy. The compound can also synergize the therapeutic effect of chloroquine by causing large-scale apoptosis [132]. Trans-chalcone is an antioxidant with anti-inflammation property, which can increase p53 activity and decrease $\beta$-catenin expression to induce an autophagy cell death in hepatocellular carcinoma (HCC) [133]. Some other compounds can also trigger autophagy through activating p53 and downstream factors of p53, such as $\beta$-asarone and 6-Azauridine [134, 135]. Moreover, BECN gene is a promising target to regulate autophagy activity which is downregulated in multiple malignancies. Oseltamivir, known as an anti-influenza virus drug, was found to upregulate BECN expression and suppress p62 expression and then cause an autophagic cell death in HCC cells [136].

A number of evidence have showed autophagic cell death is a critical event in anti-tumor effect of drugs and reverse progression of drug resistance. Targeting autophagy-induced cell death may offer new opportunities to exploit novel therapeutic strategies. As the complicated role of autophagy in cell survival and cell death, the underlying mechanism in both molecular and cellular aspects of autophagy should be further explored.

### Apoptosis

Targeting the signaling pathways to induce cell apoptosis becomes an enlightening strategy to develop novel drugs for killing cancers. Interruption of the balance of apoptosis associated signals could help induce apoptosis theoretically. The first drug applied in clinical trial and then proved to benefit patients with chronic myeloid leukemia is Oblimersen sodium, a BCL2 antisense oligonucleotide [21]. Some small molecule inhibitors of BCL2 family were also reported as entering clinical trials, such as ABT-263/navitoclax, or approved for the treatment of chronic lymphocytic leukemia like ABT-199/venetoclax, due to its BCL2 specificity and low toxicity [21]. The drugs targeting XIAP also show a clinical promise, which was combined with chemotherapy to improve the therapeutic effect in lung cancer cells in vitro and in vivo [137]. Some drugs can induce apoptosis in myeloma and acute myeloid leukemia via upregulation of BAK protein expression, for example, a phosphatase 1/6 BCI and AZD5991 [138].

P53 is a widely known tumor suppressor which is silenced in multiple types of cancers. Restoring p53 expression can enhance cell apoptosis. XI-011 and protoporphyrin IX (PpIX) can inhibit MDMX or disrupt the interaction of p53 and MDMX, consequently promote p53 transcription and trigger apoptosis in cervical cancer cells and chronic lymphocytic leukemia cells respectively [130]. The compounds of methyl β-orsellinate based 3, 5-disubstituted isoxazole hybrids can inhibit cell cycle and induce apoptosis upon p53 activation and BAX expression in breast cancer cell lines [130].

NF$\kappa$B is an important regulatory factor in the anti-apoptosis process. To modulate the activity of NF$\kappa$B can influence the balance of cell death and survival. Selinexor is an inhibitor of NF$\kappa$B, which can decrease survivin, induce apoptosis and alleviate tumor growth [139]. The phase I clinical trial of selinexor has been done in patients with advanced solid tumors and the result showed a therapeutic effectiveness and safety [140]. The combination of doxorubicin and NF$\kappa$B inhibitors in breast cancer cells can restrain multidrug resistance by blocking p65 activation, supressing cell migration and inducing typical apoptotic proteins [130].

TRAIL can specifically trigger apoptosis in tumor cells. Shishodia et al. observed that tetrandrine improved the sensitivity of prostate cancer cells to TRAIL-associated apoptosis through the upregulation of DR4/DR5 [141]. ONC201 is a TRAIL-inducing compound, which can promote the expression of TRAIL and its receptor DR5 through the activation of ATF4 by enhancing caseinolytic protease P (ClpP) [142]. It has already showed the anti-tumor effect in multiple cancer cells. Tannic acid is a natural polyphenol compound which can drive TRAIL-associated apoptosis in pluripotent embryonal carcinoma cells through upregulating the production of mitochondrial reactive oxygen species (mROS) [143].

Wnt/β-catenin signaling, JAK-STAT signaling and PI3K/Akt/mTORC1 signaling are also reported as negative regulators of cell apoptosis [130]. Once the signals are suppressed, the cell proliferation and migration should be suppressed and apoptosis can be enhanced. A series of small molecule inhibitors showed a repressive effect in various cancer cells, but there is still a gap from the bench to the bed due to lack of specificity for cancer cells and other unknown side effects. The further study is necessary to discover novel drugs with high specificity for tumor cells and novel strategy of combined therapies to overcome treatment resistance and improve patients’ responses.

### Necroptosis

As previously reported, several small molecule compounds can be capable to induce necroptosis targeting different pathways, which are promising to be developed as novel medicine for tumor therapy. The polypeptide Su-X can increase necrotic apoptosis of myeloma cells by upregulating the necroptosis-associated proteins, such as p-RIPK1/3 and p-MLKL. The mitochondrial complex I inhibitor arctigenin can boost necroptosis in prostate cancer by damaging mitochondria and enhancing the activation of RIP3 and MLKL[144]. Ophiopogonin D’ (OPD’) is a natural compound which can upregulate the expression of FasL-dependent RIPK1 and cause necroptotic cell death in metastatic prostate cancer cells [145]. Fingolimod can prompt necroptosis in lung cancer cell by binding to I2PP2A oncoprotein and then triggering the PP2A/ RIPK1 pathway [146]. Obatoclax (GX15-070), which is an antagonist of BCL proteins, was found to promote necroptotic cell death through the formation of necrosome in rhabdomyosarcoma [147, 148]. Staurosporine can trigger necroptosis by inhibiting caspase activity in leukemia cells [147, 148]. An amiloride derivative, UCD38B, was reported to act as an inhibitor of urokinase plasminogen. It can induce a caspase-independent cell death in both proliferating and non-proliferating glioma tumor cells through interruption of mitochondrial membrane and release of apoptosis-inducing factors [149]. Shikonin, a natural naphthoquinone product, was shown to increase necroptosis in apoptosis-resistant malignant cells by enhanced production of ROS in nasopharyngeal carcinoma [150].

However, the cells undergoing necroptosis were failed to induce effective adaptive immune response. It reminds that the therapy involving necroptosis-targeting agents should be designed carefully due to the complicated regulation of tumor and tumor microenvironment. The studies on necroptosis-related therapies are mostly in the bench side. Future pre-clinical experiments and clinical trials are remained to be well-conducted.

### Pyroptosis

An increasing number of reports have showed the potential of targeting pyroptosis in tumor therapy, which can not only cause cell death, but also lead to an antitumor immune response. It was reported that methotrexate was packaged in microparticles released by tumor cells and delivered to cholangiocarcinoma cell and induce pyroptosis, subsequently macrophages and neutrophils were activated and recruited to tumor site [151]. Metformin, known as a drug for diabetes, was proved to induce pyroptosis in multiple tumor cells through a caspase-dependent manner [152]. Ivermectin is an FDA-approved antiparasitic drug and allosterically regulates P2X4 receptors. It was applied in breast cancer cells and drived pyroptosis by opening the P2X4/P2X7-gated pannexin-1 channels [153]. The inhibition of BRD4 can result in a caspase1/GSDMD dependent pyroptosis in renal carcinoma cells [154]. A novel anti-tumor drug, $\alpha$-NETA, can cause pyroptosis in ovarian cancer cell lines and tumor-bore mice via GSDMD/caspase-4 pathway [155]. The compound L61H10 showed the anti-tumor activity in lung cancer by the switch of apoptosis and pyroptosis harnessing the NF$\kappa$B signaling pathway [156]. Dihydroartemisinin which is a derivative of artemisinin extraction can activate caspase-3, upregulate DFNA5 and AIM2 expression and ultimately promote pyroptosis in breas cancer cells [157]. Chimeric antigen receptor gene-modified T (CAR-T) cells can also trigger GSDME-mediated pyroptosis in leukemia, which suggests a synergistic effect of cell death and immunotherapy [104].

### Ferroptosis

During recent years, ferroptosis has drawn considerable interest due to its therapeutic potential in tumor. Increasing evidence shows ferroptosis participates in tumor suppressive progression induced by multiple conventional therapy strategies, thus it is promising to discover potential drug candidates to enhance ferroptosis in tumor as novel therapeutic methods [158].

RSL3 is a ferroptosis activator, which inhibits GPX4 and promotes the accumulation of ROS. It has been proved to cause lethality in cancer cell lines in vitro and inhibit tumor growth in vivo. The synthetic analogs of RSL3, like chloroacetamide and chloromethyltriazine compounds, can also covalently bind to GPX4 and inhibit its activity[104]. Another ferroptosis inducer is erastin, which can decrease GSH synthesis by directly inhibiting cystine/glutamate antiporter system Xc- [159]. The downstream molecular targets of erastin includes RAF/MEK/ERK signaling pathway and mitochondrial VDAC to improve the accumulation of ROS. In addition, erastin can facilitate the conventional chemotherapy effect in several cancer cell lines, such as glioblastoma (GBM), acute myeloid leukemia (AML) and head and neck cancer [160]. Sulfasalazine is broadly applied in the treatment of chronic inflammation. The drug can inhibit NF$\kappa$B signaling as well as disrupt the transporter Xc-, which is like erastin as a manner to induce ferroptosis in different malignancies, such as AML, HCC and ovarian clear cell carcinoma [161]. Sorafenib drives ferroptosis in certain cancer cell lines including HCC, renal cell carcinoma and thyroid tumor by inhibiting GSH production and the system Xc- activity [109]. Statins, such as fluvastatin, lovastatin and simvastatin, are used as the inhibitor of HMGCR. The drug promotes ferroptosis through reducing GPX4 or CoQ10 production [162]. Several clinical trials have verified atorvastatin and fluvastatin could retard cell proliferation in the tumor overexpressing HMGCR [162]. Lovastatin can transform a “cold” tumor into an inflammatory phenotype by inducing ferroptosis in non-small cell lung cancer (NSCLC). It also decreases the expression of PD-L1 to improve the susceptibility of tumor cells to ICB therapy [163].

Besides malignant cells, the other cell types in TME are also susceptible to ferroptosis. Therefore, it is critical to take the ferroptosis vulnerabilities of tumor cells and immune cells together into consideration to achieve a probably balance and a better therapy effect.

### Cuproptosis

With the emergence of cuproptosis, the approaches to manipulate copper ions to induce cell death are promising to become novel tumor therapies. Copper ionophores refer to the chemicals or compounds which are capable to elevate the intracellular copper levels [164]. Disulfiram (DSF), a copper-binding compound, can be applied together with copper to induce cuproptosis and reduce tumor growth [165]. The disulfiram-copper compounds were also found to help overcome chemotherapy resistance in oral cancer cells [166]. NSC319726 was reported as a specific inhibitor of tumors carrying p53^R175H^ mutant previously. It has been noticed recently that the chemical is a copper ionophore which can lead to cell death through binding with copper and triggering cuproptosis [167]. The drug was applied in tumor cells of glioblastoma patients and showed a tumor-suppressive effect. Curcumin, a natural copper ionophore, was proved to induce cuproptosis in colorectal cancer (CRC) cells with a good efficacy and safety [168]. Copper chelators can also promote the accumulation of copper in cells. In addition, those compounds can exert a role in PD-L1 degradation, inhibiting cell proliferation and tumor growth and improving anti-tumor immune response. For example, tetrathiomolybdate and triethylenetetramine can reduce the growth and metastasis of various preclinical models of mesothelioma, pancreatic adenocarcinoma, ovarian carcinoma, melanoma and CRC [169, 170]. The combination of tetrathiomolybdate and other drugs like doxorubicin, cisplatin and 5-fluorouracil exhibited a synergistic effect to enhance the cytotoxicity in CRC, cervical and ovarian cancer cell lines [167].

Tumors with strong mitochondrial metabolism could be more sensitive to cuproptosis. To combine cuproptosis inducers with drugs causing a high level of mitochondrial metabolism can be a potential innovative therapeutic approach. Aerobic glycolysis is the primary method to produce energy for cells within tumors. 4-Octyl itaconate can reduce the level of aerobic glycolysis in CRC cells and improve the susceptibility of tumor cells to cuproptosis, which harbors the potency to elevate the inhibitory effect of aerobic glycolysis-targeted treatment [171].

Some nanoparticles (NPs) can also induce cuproptosis to show the potency as anti-tumor drugs. The HD/BER/GOx/Cu hydrogel system release DSF thereby increase the copper level to induce apoptosis and cuproptosis. The NPs have showed the capability to inhibit tumor growth and invasion in breast cancer [172]. NP@ESCu can trigger cuproptosis and enhance immune response by the production of elesclomol and copper, which has been validated in tumor cells and animal models of bladder cancer to show an anti-tumor effect with PD-L1 blockade [173]. CuET NPs are found to reverse cislpatin resistance in NSCLC cells through improving cuproptosis with good anti-tumor activity and biosafety [174].

Cuproptosis inducers can be effective anti-tumor agents, while their side effects should be further investigated due to various metabolic states of different organs. It is also important for developing cuproptosis-based therapies to improve the safety and achieve a better response.

## Alterations induced by cell death in tumor microenvironment

Over the years, cancer has been acknowledged as an evolutionary and ecological phenomenon, characterized by co-evolution of cancer cells and the surrounding microenvironments [175]. Both tumor cell and tumor microenvironment are dynamically altered during multiple tumor-associated events, like tumorigenesis, progression, metastasis, relapse and response or resistance to treatments. Genetic and epigenetic changes in the tumor cells, along with the remodeling of the components of the TME, result in the tumor heterogeneity, varied clinical outcomes and therapy responses among patients [176].

**Fig. 7:**
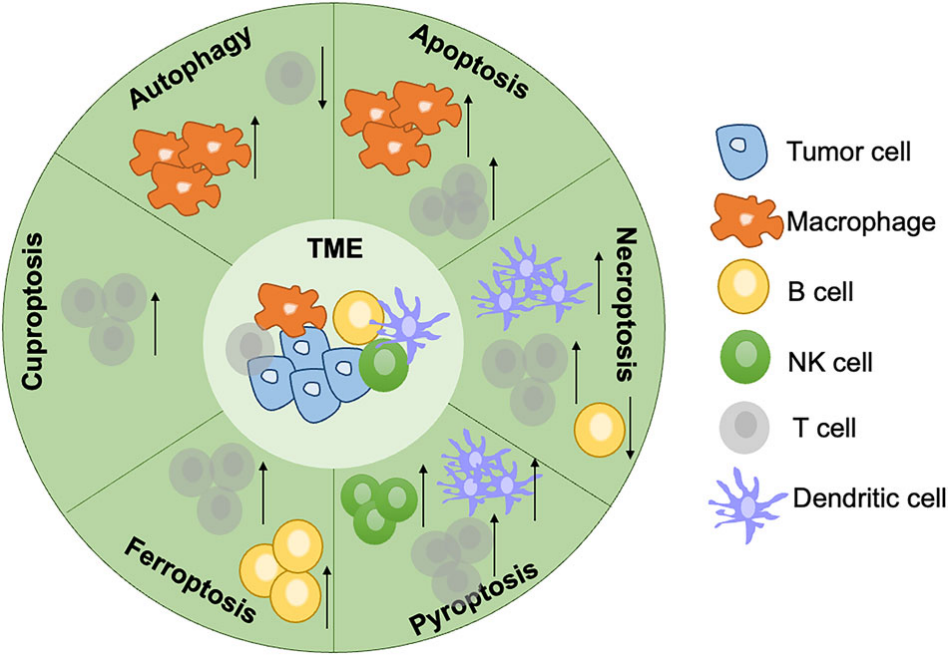
The effects of RCDs in tumor microenvironment. The effects of autophagy, apoptosis, necroptosis, pyroptosis, ferroptosis, and cuproptosis on the tumor microenvironment were summarized.

Cell death can be caused by multiple cancer therapy methods. It is a downstream process induced by chemotoxicity or physical injury from drugs or radiation. Tumor cells can employ the blockade of cell death to survive from therapies and it is necessary to explore the TME changes to gain additional views to understand how the cells escape from programmed death and immune surveillance (Fig. 7).

### Autophagy

There are contrary descriptions of the influence of autophagy in tumor cells on anti-tumor immunity [129]. The induction of tumor cell autophagy after chemotherapy is necessary for immunogenic cell clearance and maintaining antitumor immune cell infiltration. To restrict calorie intake before anti-tumor drug administration is also an efficient method to improve autophagy in tumor cells so as to enhance immunosurveillance and decrease tumor burden [129]. Whereas the anti-tumor immune responses can be inhibited due to the declined secretion of CCL5 by autophagic tumor cells, which can cause a recruitment of NK cells. The abundant nutrients derived from autophagic cells can support the survival of not only malignant cells but also other immune or stromal cells in TME [129]. The autophagy in cancer-associated fibroblasts were reported to promote pancreatic tumor progression via the production of IL6. Autophagy in CD8^+^ T cells can reduce IFN$\gamma$ level and inhibit memory effector phenotype skewing [177]. As given the distinct roles of autophagy in TME components, it is important to synthetically evaluate the features of immune and metabolism in TME upon certain treatment resistance to gain a more comprehensive view of the relevant mechanism.

### Apoptosis

Apoptosis is known as a non-immunogenic programmed cell death, but the signals triggered during apoptosis can further influence the behaviors of other components in tumor microenvironment. The cells undergoing apoptosis produce some chemoattractant factors and proliferative signals to force the TME to clearing and regenerating. For example, the purine nucleotides released from apoptotic cells could recruit the phagocytes including macrophages and dendritic cells (DCs). The sphingosine-1-phosphate (S1P) and endothelial monocyte-activating protein (EMAP) secreted by apoptotic cells were able to remodel the stroma component [178]. The stimulation and maturation of dendritic cells can be inhibited by the cell engulfment induced by apoptosis, result in that the antigen presentation is blocked and effective immune response cannot be activated [179]. When macrophages or DCs undergo apoptosis after phagocytizing apoptotic cells, the production of immunosuppressive factors was observed and helped shape the TME as immunosuppressive with expansion of regulatory T cells [179]. In addition, the acidic environment, hypoxia and high level of ROS in TME could facilitate the survival of immunosuppressive immune cells such as regulatory T cells (Tregs), M2 macrophages and MDSCs. The apoptosis of cytotoxic immune cells would impair the anti-tumor immunity. Thus, it is promising to assess different methods of the induction of apoptosis in TME to achieve an outcome of tumor cell elimination and anti-tumor immunity retaining.

### Necroptosis

The immunogenicity of necroptotic cancer cells increases not only the tumor antigen burdens but also the anti-tumor immunity [146]. In one way, necroptosis could enhance the DC maturation and the adaptive immunity priming, in the other way, necroptosis might play a role in promoting the immunosuppressive conditions [146]. RIPK3 acts as an important player in modulation of TME. It was reported that the elimination of RIPK3 decreases the infiltration of MDSCs and tumor-associated macrophages (TAMs) and increases the proportions of lymphocytes, indicating an immune activated context [179]. In contrary, RIPK3 deletion in macrophage force the M2 phenotype (anti-inflammation) polarization, which further facilitate tumor progression [146]. It is an effective way to vaccinate the tumor with necroptotic cancer cells to activate innate and adaptive immunity against tumors. Transfection of RIPK3 into tumor cells can induce necroptosis, which can be synergized with immunotherapy to maintain an activated immune response. The proinflammatory factors within TME are also increased, resulting in a cytotoxic effect. The inflamed TME altered by necroptosis induction improves the susceptibility of tumor to immunotherapy [179, 180].

Moreover, the necroptotic cells can modulate TME via different cellular contents released during necroptosis, such as the vascular endothelial growth factor (VEGF) and fibroblast growth factor (FGF) which act as pro-angiogenic factors [181]. It was also reported that necroptosis could influence tumor blood vessel formation and maintenance. The matrix metalloproteinases (MMPs) derived from necroptotic cells can remodel the TME to facilitate the invasion and metastasis of tumor cells [181]. It is necessary to surveillance and interpret the TME alterations carefully caused by necroptosis to help obtain a comprehensive view and identify novel promising therapeutic targets.

### Pyroptosis

Pyroptosis plays a critical role in regulating the TME, being involved in both tumorigenesis and antitumor immune responses throughout various stages of tumor progression. The effects of pyroptosis on tumors, whether promoting or suppressing, primarily depend on various factors including the tumor type, host inflammatory status, immune response, and the specific effector molecules involved. Pyroptosis cell death usually induce inflammation and production of pro-inflammatory cytokines like IL-1$\beta$ and IL-18, which help enhance the innate immunity and recruit effector adaptive immune cells into TME to lead to an immune-activated TME [179]. For instance, IL-1$\beta$ released from pyroptotic cells can drive DC maturation and monocyte differentiation. It also can directly activate the cytotoxic T cells and promote Th1 cell infiltration. HMGB1 produced by the pyroptotic tumor cells can promote the APC activation and antigen presentation. Further it can bind to TLR2 or TLR4 and subsequently result in the upregulation of NF$\kappa$B and AP-1 signaling, the increased production of TNF$\alpha$, IL-6, IL-1, IL-18 and other co-stimulatory molecules for cross-priming of cytotoxic T cells [182]. However, HMGB1 enhanced by pyroptosis could further activate ERK pathway, which is a signaling participated in tumor progression through polarizing macrophage and resulting in an immunosuppressive TME [183].

The PARPi treatment can enhance GSDMC mediated pyroptosis and GSDMC can sensitize tumor cells to PARPi via an immune-dependent manner, which forms a positive feedback loop to achieve a better treatment response [184]. GSDMC induced pyroptosis in tumor cells can restore the infiltration of memory T cells in spleen, lymph node and TME, and then increase the cytotoxic T cell. GSDME expressed by the pyroptostic cell the combined treatment of BRAF and MEK inhibitors in melanoma can elevate the amount of T cells and DCs, while GSDME-deficient melanoma exhibited less HMGB1 release and BRAFi and MEKi resistance [185]. However, the other animal study showed that long-term chronic cell death might promote tumor growth and GSDMC expression was correlated with PD-1 expression and poor clinical outcomes in triple-negative breast cancer patients [186].

Pyroptostic tumor cells are immunogenic which can trigger anti-tumor immunity and facilitate the immunotherapy. Thus, it is promising to combine the pyroptosis induction and TME remodeling to boost the effect of immunotherapy, elongate the antitumor immune response and inhibit tumor metastasis and relapse.

### Ferroptosis

The effect of ferroptosis on TME can be dependent on the different ferroptotic stages. The tumor cells initiating ferroptosis can be immunogenic to stimulate a vaccination-like response and recruit the myeloid cells to eliminate the dying cells [187]. During the late ferroptosis, PGE2 is released to play a role in suppression of anti-tumor cells like natural killer (NK) cells and cytotoxic T cells [146]. The oxidized lipids released from ferroptotic cells can also inhibit DC maturation to facilitate tumor cells to escape from immune surveillance [188]. It was demonstrated that PGE2 could inhibit the recruitment of DCs into tumor via decreasing CCL5 and XCL1 produced by NK cells, which suggested ferroptosis could impair pro-inflammatory immunity through affecting on innate immune [189]. In addition, the immune cells in TME show various levels of sensitivity to ferroptosis, for example, M2 macrophages harbor a higher sensitivity than M1 macrophages [190]. It is potential to develop a therapy targeting diminishing M2 macrophages with induction of ferroptosis to overcome immunosuppression in TME.

KRAS^G12D^ is the most common mutant of KRAS oncogene, which can be secreted by the ferroptotic cancer cell as exosomes into TME and then be recognized by macrophages. KRAS^G12D^ can drive the macrophage polarization and boost tumor growth. HMGB1 produced by the ferroptotic tumor cells can also promote M1 polarization through the HMGB1-AGER interaction [191].

Ferroptosis has been reported to exert a role in T lymphocyte immunity. The loss of glutathione peroxidase 4 (GPX4) in T cells can promote ferroptosis and targeting to eliminate GPX4 in Tregs can attenuate tumor growth and enhance anti-tumor immunity. Anoctamin 1 (ANO1) can improve the recruitment of cancer-associated fibroblasts (CAFs) into TME by restraining tumor ferroptosis and promoting TGF$\beta$ secretion, which is a potential mechanism of drug resistance [179, 192]. The high level of ferroptosis is associated with neutrophil terminally differentiation. CXCR4^+^ neutrophils are immunosuppressive subtype with a ferroptotic phenotype whereas ACOD1^+^ neutrophils are resistant to ferroptosis and can stimulate tumor metastasis [192]. Due to the complicated role of ferroptosis in various components within TME, more efforts should be input to learn how the process alter tumor context in both immune and metabolic ways.

### Cuproptosis

It is known that Cu concentrations in blood and tissue are positively correlated to tumor proliferation and metastasis [193]. Recent studies found that the cellular copper level could affect PD-L1 expression to help tumor cell evade from immunosurveillance, thus it is reasonable that cuproptosis can play a role in modulating tumor progression events via remodeling the TME [169]. It was reported that cuproptosis could enhance anti-tumor immunity through cGAS-STING pathway [164]. When inducing cuproptosis in clear cell renal cell carcinoma (ccRCC) cells, the co-cultured DCs harbored an increased cGAS-STING expression. The concentrations of some pro-inflammatory factors, such as IL-2, TNF$\alpha$, IFN$\gamma$, CXCL10 and CXCL11, derived from the co-culture system were also improved. It has been proved in animal models that the circulating CD8^+^ T cells were elevated when combining cuproptosis inducing with anti-PD1 therapy [164].

A previous study in oral squamous cell tumor showed arecoline could downregulate cuproptosis and then upregulate the viability of CAFs, which contributed to tumor metastasis and drug resistance [166]. In addition, cuproptosis-predominant tumors exhibited declined angiogenesis and increased susceptibility to certain treatments. The key regulator of cuproptosis, FDX1, can be related to tumor metastasis stages and patient outcomes. The high expression level of FDX 1 can predict a long survive time verified in multiple tumor types [146]. The other cuproptosis-related genes were also reported to be correlated with PD-L1 expression and immune cell infiltration, but more cellular or animal experiments should be performed to validate the results upon the gene set scoring method [146].

## Application of high-throughput sequencing technologies in cell death study

As the application of omics data is popularized in the investigations of potential mechanisms of biological and medical events, more factors in the levels of non-coding RNAs and small RNAs are involved in cell death in addition to classical molecular mechanisms. The stratification of patients based on the expression of gene signatures related to cell death can be applied in prognosis predictions and treatment decisions theoretically.

### Autophagy

Autophagy is widely associated with various events in tumor progression. A risk-predictive model based autophagy was built through integrative analysis of bulk RNA sequencing (RNA-seq) and single-cell RNA sequencing data (scRNA-seq) [194]. The model could be applied in stratify gastric cancer (GC) patients with their prognosis, which was further validated in TCGA cohort and two independent GEO cohorts using survival analysis. The utilization of single-cell RNA-seq data suggested the model could indicate a dysfunctional T cell phenotype. In addition, the high-risk score of autophagy was associated with a lower expression of PDCD1 and CTLA4, which might hold the potency to predict the immunotherapy response of GC patients. GBM is the most common malignant primary brain cancer in adults, characterized by high aggressiveness and a poor prognosis [195]. MGCG is a long non-coding RNA (lncRNA) closely associated with tumorigenesis, progression, and prognosis in various cancers. The expression of MGCG in GBM cells was analyzed using high-throughput RNA-seq technology [196]. MGCG was found to promote autophagy and exacerbate GBM tumor formation by regulating the expression of ATG2A. Further mass spectrometry analysis revealed that MGCG directly interacts with the hnRNPK protein. The MGCG/hnRNPK complex enhances the translation of ATG2A, thereby facilitating the autophagy process. These findings suggest that MGCG may serve as a novel target for the molecular diagnosis and treatment of GBM. To further investigate the features of autophagy in TME by high-throughput sequencing technology could provide a solid theoretical foundation for the development of future immunotherapy strategies targeting autophagy.

### Apoptosis

Apoptosis is not associated with the release of significant amounts of inflammatory factors or DAMPs, which makes apoptotic cells less likely to provoke an immune response. Consequently, the expression of apoptosis-related genes is typically weak in scRNA-seq data, which confuses the differentiation between dead and viable cells. Furthermore, apoptotic cells are rapidly engulfed by surrounding macrophages and other phagocytes after death, resulting in a brief presence in tissues, further limiting their detection by single-cell RNA sequencing. In contrast, cell death mechanisms such as pyroptosis and necrosis lead to cell rupture and the release of cellular contents, which in turn trigger immune responses, making dead cells more readily identifiable and detectable. Although the molecular mechanisms of apoptosis have been extensively studied, and the related genes and pathways (e.g., caspase signaling, BCL2 family) are well-established across various cell death modalities, with the advancement of new technologies, apoptosis research may see a resurgence, particularly through the integration of emerging approaches such as spatial transcriptomics and caspase activity markers, which may offer deeper insights into apoptosis mechanisms.

### Necroptosis

Necroptosis, an emerging area of interest in cell death research, has garnered significant attention in the context of cancer. Zhao et al. applied the Least Absolute Shrinkage and Selection Operator (LASSO) technique to comprehensively analyze information from 204 normal samples and 343 tumor samples and to construct the necroptosis-related lncRNA model [197]. The model’s verification and evaluation were conducted through Kaplan-Meier analysis, time-dependent receiver operating characteristic (ROC) analysis, univariate Cox (uni-Cox) regression, multivariate Cox (multi-Cox) regression, nomogram creation, and calibration curve plotting. Necroptosis-associated lncRNAs hold the capacity not only to prognosticate outcomes but also to discern cold and hot tumor profiles in gastric cancer [197]. In a related study, Wang et al. indicated a general upregulation of most necroptosis regulators, with notable downregulation of TLR3, ALDH2, and NDRG2 mRNA levels in gastric cancer [198]. Analyses of these regulators highlighted their involvement in programmed necrotic cell death, along with crucial pathways like TNF signaling, NF-$\kappa$B signaling, and NOD-like receptor signaling. They identified lncRNA SNHG1/miR-21-5p/TLR4 regulatory axis which involves lncRNA SNHG1’s inhibition of cell proliferation and promotion of apoptosis, mediated by miR-21-5p and TLR4 downregulation [198]. However, these findings lack further validation through in vitro and in vivo experiments. Furthermore, Zhao et al. selected 29 necroptosis-related genes to categorize patients into distinct necroptosis phenotypes through unsupervised consensus clustering methods. Additionally, the authors classified 1064 lung adenocarcinoma patients into 3 molecular phenotypes by analyzing the expression levels of necroptosis related molecules and developed a new scoring system named “NecroScore” to quantify the degree of necroptosis in tumors and predict patients’ survival outcomes [199]. The sequencing data can provide more comprehensive information to discover novel molecular factors participated in regulation of cell death and to classify patients into accurate molecular subtypes combined with clinical features. Those findings may help develop novel therapy methods, novel predictive markers and prognostic models for disease stages, drug sensitivity or patients’ survival.

### Pyroptosis

In a ground-breaking study, Ye et al. conducted an investigation into the mRNA expression levels of 33 presently recognized pyroptosis-related genes across 88 normal and 379 tumor tissues, revealing significant differential expression among them [200]. The 379 ovarian cancer patients were categorized into low- and high-risk subgroups according to the median risk score of a 7-gene signature derived from pyroptosis-related genes. Within the TCGA cohort, the high-risk subgroup generally exhibited diminished immune cell infiltration. Additionally, the research unveiled that the infiltrations of DCs, induced dendritic cells (iDCs), and macrophages were enriched, while type-2 IFN responses were attenuated in the low-risk group compared to the high-risk group[200]. Meanwhile, Zhang et al. classified muscle-invasive bladder cancer (MIBC) into three pyroptosis patterns, pyroptosis activation (Cluster 1), pyroptosis inactivation (Cluster 2), and moderate pyroptosis activation (Cluster 3) through quantifying the expression levels of GSDMB and 10 canonical cleavage enzymes related to pyroptosis in 909 MIBC samples with transcriptomic data obtained from TCGA database and GSE87304, GSE31684, GSE48075 and GSE169455 cohorts [201]. By performing PCA on the 57 differentially expressed genes, they formulated an innovative predictive model termed the pyroptosis-related gene score (PRGScore) to measure the pyroptosis status of individual cases of MIBC [201]. Furthermore, the samples from the TCGA-BC dataset and GSE20685 dataset were divided into three pyroptosis subtypes based on the expression of the 40 genes, which offer direction for personalized assessment and treatment decisions [202]. The molecular subtypes based on different pyroptosis-associated genes can stratify the patients with their immune status to facilitate the clinical decisions of immunotherapy in addition to the correlation with patients’ prognosis.

### Ferroptosis

In the pioneering work, Li et al. utilized gene set variation analysis (GSVA) to analyze the ferroptosis scores and immune scores in the TARGET-OS dataset [203]. They subsequently classified and constructed a co-expression network using weighted gene co-expression network analysis (WGCNA) based on these scores. This led to the identification of a gene set comprising 327 ferroptosis-related candidates and 306 immune-related candidates. Using LASSO-Cox regression analysis, they evaluated a 4-gene signature (WAS, CORT, WNT16, and GLB1L2) to predict overall survival (OS) [203]. Fan et al. identified 9 differentially expressed ferroptosis-related genes (PR-DE-FRGs) from 5 cohorts, including TCGA, GEO, ICGC and FerrDb database [204]. They constructed a prognostic model and validated it across 6 datasets using Kaplan–Meier curves, ROC curves and PCA. Subsequently, they delved into the multifaceted role of ferroptosis in oral squamous cell carcinoma (OSCC), considering signaling pathways, immunity, mutations, and cellular stemness. Simultaneously, Wu et al. obtained data from 437 colon adenocarcinoma (COAD) samples and the sequences of ferroptosis-related genes (FRGs) in Homo sapiens from the TCGA database and FerrDb databases [205]. They identified 14086 lncRNAs and 176 FRGs and employed spearman correlation analysis to identify 705 ferroptosis-related lncRNAs (FRLs). Univariate COX analysis was then used to screen the prognostic FRLs. Subsequently they constructed a 4-FRL signature through optimal penalty parameter (λ) selection for the LASSO model. The prognostic ability and potential function of the model were evaluated using Kaplan-Meier survival curve analysis, ROC curve analysis, gene set enrichment analysis (GSEA), immune-related analysis, somatic mutation analysis, and drug sensitivity assays.

The emergence of sequencing technologies and bioinformatic tools has enabled the utilization of single-cell analysis to reveal the extraordinary intricacy of TME [206, 207]. The single cell sequencing is revolutionizing our understanding across different cancer types, as well as providing novel insights into the heterogeneity, plasticity, and functional diversity of the immune and stroma compartments in tumor. The unique signatures of the cellular components, the associated signaling and the diversity of TMEs, have been targeted in cancer therapy. It reminds us that the features of cell death can be dissected and classified according to the diverse cell composes and behaviors in TME, which can serve as certain novel elements to stratify patients with different survival outcomes or therapy responses.

### Cuproptosis

High-throughput sequencing technology is used to identify potential targets and pathways associated with cuproptosis, while in vitro and in vivo experiments are employed to verify the biological functions of th ese targets. This combined approach not only enhances the reliability of the results but also increases the potential for clinical translation. Zhao et al. integrated scRNA-seq and bulk RNA-seq data to analyze the regulatory patterns of cuproptosis in the sepsis immune microenvironment [208]. By analyzing the scRNA-seq data, they mapped the immune microenvironment of sepsis patients, including immune cell composition and activity, and further evaluated the association between the cuproptosis activity score (CuAS) and immune microenvironment characteristics, such as immune cell infiltration and the expression of inflammatory factors. Key cuproptosis-related genes, including LST1, MMP9, IL1RN, CXCL10, and TYROBP, were identified using the WGCNA algorithm, LASSO regression, and Cox regression analysis. These genes were subsequently used to construct a riskScore model. The predictive power of this model was validated using multiple independent GEO datasets, and a nomogram combining risk scores with clinical features was designed to evaluate its clinical utility in sepsis prognosis. Similarly, Yang et al. used RNA sequencing data from HCC patients in the TCGA database to construct a prognostic model based on cuproptosis-related genes [209]. They employed LASSO Cox regression analysis, immune infiltration analysis, single-cell RNA sequencing technology, and data from 116 HCC samples in their internal cohort to validate that the polygenic risk scoring model based on cuproptosis-related genes (CRGs) demonstrated good predictive performance across different cohorts. The model effectively distinguishes between high-risk and low-risk patients, with the high-risk group showing significantly worse prognosis. Kaplan–Meier curve analysis and ROC curve analysis confirmed the consistency and reliability of the risk scoring model across cohorts. Immune cell infiltration analysis of HCC samples revealed that the immune microenvironment characteristics associated with cuproptosis were reproducible in the validation cohorts, particularly the enrichment of regulatory T cells and macrophages in cuproptosis-related HCC samples. The enrichment of this immune infiltration signature correlates with the depletion of T cell proliferation, further supporting the potential role of cuproptosis in immune escape and tumor progression. The concentration of copper ions in the serum of cancer patients is higher than that in healthy individuals and is closely associated with disease progression and treatment [210]. Zhao et al. downloaded RNA sequencing datasets and clinical data from tumor tissues of 371 liver cancer patients and 50 normal liver tissues from TCGA database [211]. They extracted the expression matrices of 10 known genes related to cuproptosis and used the Wilcoxon rank-sum test to analyze the differential expression between groups. In the TCGA cohort, among the 10 cuproptosis-related genes associated with tumor and normal tissues, only FDX1 was downregulated in HCC tissues, while the expression levels of the other genes were upregulated. LASSO regression and Cox regression analysis were employed to construct a prognostic risk scoring model based on CRGs, which effectively distinguished high-risk and low-risk patients and provided a reference for the early diagnosis and prognosis of liver cancer. The FDX1 expression in tumor cells was also positively related to CD8 T cell proportion and negatively associated with immunosuppressive molecules, which indicates FDX1-involved cuproptosis can trigger the antitumor immunity. A bunch of bioinforamtic studies has proved cuproptosis-based gene features can not only improve the knowledge of certain cancer biology, but also provide a clinical translational potency applied in prediction of patients’ survival or treatment evaluation.

## Prospective

Traditionally the drug target discovery could be dependent on genome and RNA information. The potential novel therapeutic targets can be derived from homologs of gene families with known targets. The search for novel targets is based on the genome sequence and protein information [212]. Comparing RNA profiles, between tumor and normal tissues, or between tissues with drug treatment and tissues without, can be also applied in drug target discovery. The outcome usually provides tens of genes, therefore further characterization and validation should be conducted to identify biomarkers or therapeutic targets.

There has been an increase of significance of dissect the complicated cellular talks in TME to understand the dynamic alterations of tumor cell and other components in TME during immunogenic cell death and investigate potential novel therapeutic targets. Novel insights of cell death mediated drug resistance and therapeutic target identification in TME are emerging due to the comprehensive cell subtyping and molecular feature by single cell sequencing [213]. When comparing TMEs between before and after treatment groups, or before and after resistance group, single cell RNA sequencing is a powerful tool to characterize new targets with the potency of reactivating immune response and relieving drug resistance from the reshaped TMEs [206, 213]. In order to improve the credibility and priority of the identified targets, other multiplex sequencing data can be incorporated with scRNA-seq.

Genome-wide association studies (GWAS) is widely applied in discovering genetic variants associated with disease according to abundant human genetic data. If integrating the gene expression in specific cell type and the gene variant found from the same cohort by GWAS, it will be possible to screen novel effector genes to be participated in cell death, to predict disease risk and to act as novel therapy approaches in a single cellular level [5, 212].

## References

[1] Hotchkiss RS, Strasser A, McDunn JE, Swanson PE. Cell death. N. Engl J Med. 2009;361:1570–83. 19828534 10.1056/NEJMra0901217 PMC3760419

[2] Green DR. Cell death in development. Cold Spring Harb Perspect Biol. 2022;14:a041095. 10.1101/cshperspect.a041095 PMC9121905 35581003

[3] Newton K, Strasser A, Kayagaki N, Dixit VM. Cell death. Cell. 2024;187:235–56. 38242081 10.1016/j.cell.2023.11.044

[4] Bunz F. Cell death and cancer therapy. Curr Opin Pharm. 2001;1:337–41. 10.1016/s1471-4892(01)00059-5 11710730

[5] Raschella G, Melino G, Gambacurta A. Cell death in cancer in the era of precision medicine. Genes Immun. 2019;20:529–38. 30341419 10.1038/s41435-018-0048-6

[6] Strasser A, Vaux DL. Cell death in the origin and treatment of cancer. Mol Cell. 2020;78:1045–54. 32516599 10.1016/j.molcel.2020.05.014

[7] Dikic I, Elazar Z. Mechanism and medical implications of mammalian autophagy. Nat Rev Mol Cell Biol. 2018;19:349–64. 29618831 10.1038/s41580-018-0003-4

[8] Deter RL, De Duve C. Influence of glucagon, an inducer of cellular autophagy, on some physical properties of rat liver lysosomes. J Cell Biol. 1967;33:437–49. 4292315 10.1083/jcb.33.2.437 PMC2108350

[9] Tsukada M, Ohsumi Y. Isolation and characterization of autophagy-defective mutants of Saccharomyces cerevisiae. FEBS Lett. 1993;333:169–74. 8224160 10.1016/0014-5793(93)80398-e

[10] Ke PY. Horning cell self-digestion: autophagy wins the 2016 Nobel Prize in Physiology or Medicine. Biomed J. 2017;40:5–8. 28411883 10.1016/j.bj.2017.03.003 PMC6138592

[11] Kim J, Kundu M, Viollet B, Guan KL. AMPK and mTOR regulate autophagy through direct phosphorylation of Ulk1. Nat Cell Biol. 2011;13:132–41. 21258367 10.1038/ncb2152 PMC3987946

[12] Backer JM. The regulation and function of class III PI3Ks: novel roles for Vps34. Biochem J. 2008;410:1–17. 18215151 10.1042/BJ20071427

[13] Mizushima N. Autophagy: process and function. Genes Dev. 2007;21:2861–73. 18006683 10.1101/gad.1599207

[14] Kirkin V, McEwan DG, Novak I, Dikic I. A role for ubiquitin in selective autophagy. Mol Cell. 2009;34:259–69. 19450525 10.1016/j.molcel.2009.04.026

[15] Denton D, Kumar S. Autophagy-dependent cell death. Cell Death Differ. 2019;26:605–16. 30568239 10.1038/s41418-018-0252-y PMC6460387

[16] Galluzzi L, Vitale I, Aaronson SA, Abrams JM, Adam D, Agostinis P, et al. Molecular mechanisms of cell death: recommendations of the Nomenclature Committee on Cell Death 2018. Cell Death Differ. 2018;25:486–541. 29362479 10.1038/s41418-017-0012-4 PMC5864239

[17] Klionsky DJ, Petroni G, Amaravadi RK, Baehrecke EH, Ballabio A, Boya P, et al. Autophagy in major human diseases. EMBO J. 2021;40:e108863. 34459017 10.15252/embj.2021108863 PMC8488577

[18] Jung S, Jeong H, Yu SW. Autophagy as a decisive process for cell death. Exp Mol Med. 2020;52:921–30. 32591647 10.1038/s12276-020-0455-4 PMC7338414

[19] Kerr JF, Wyllie AH, Currie AR. Apoptosis: a basic biological phenomenon with wide-ranging implications in tissue kinetics. Br J Cancer. 1972;26:239–57. 4561027 10.1038/bjc.1972.33 PMC2008650

[20] Li P, Nijhawan D, Budihardjo I, Srinivasula SM, Ahmad M, Alnemri ES, Wang X. Cytochrome c and dATP-dependent formation of Apaf-1/caspase-9 complex initiates an apoptotic protease cascade. Cell. 1997;91:479–89. 9390557 10.1016/s0092-8674(00)80434-1

[21] Wong RS. Apoptosis in cancer: from pathogenesis to treatment. J Exp Clin Cancer Res. 2011;30:87. 21943236 10.1186/1756-9966-30-87 PMC3197541

[22] Thornberry NA, Lazebnik Y. Caspases: enemies within. Science. 1998;281:1312–6. 9721091 10.1126/science.281.5381.1312

[23] Green DR. Apoptotic pathways: paper wraps stone blunts scissors. Cell. 2000;102:1–4. 10929706 10.1016/s0092-8674(00)00003-9

[24] Strasser A, Harris AW, Huang DC, Krammer PH, Cory S. Bcl-2 and Fas/APO-1 regulate distinct pathways to lymphocyte apoptosis. EMBO J. 1995;14:6136–47. 8557033 10.1002/j.1460-2075.1995.tb00304.x PMC394738

[25] Czabotar PE, Lessene G, Strasser A, Adams JM. Control of apoptosis by the BCL-2 protein family: implications for physiology and therapy. Nat Rev Mol Cell Biol. 2014;15:49–63. 24355989 10.1038/nrm3722

[26] Bock FJ, Tait SWG. Mitochondria as multifaceted regulators of cell death. Nat Rev Mol Cell Biol. 2020;21:85–100. 31636403 10.1038/s41580-019-0173-8

[27] Liu Z, Sun C, Olejniczak ET, Meadows RP, Betz SF, Oost T, et al. Structural basis for binding of Smac/DIABLO to the XIAP BIR3 domain. Nature. 2000;408:1004–8. 11140637 10.1038/35050006

[28] Krammer PH. CD95’s deadly mission in the immune system. Nature. 2000;407:789–95. 11048730 10.1038/35037728

[29] Nagata S. Apoptosis by death factor. Cell. 1997;88:355–65. 9039262 10.1016/s0092-8674(00)81874-7

[30] Strasser A, Jost PJ, Nagata S. The many roles of FAS receptor signaling in the immune system. Immunity. 2009;30:180–92. 19239902 10.1016/j.immuni.2009.01.001 PMC2956119

[31] Chaudhary PM, Eby M, Jasmin A, Bookwalter A, Murray J, Hood L. Death receptor 5, a new member of the TNFR family, and DR4 induce FADD-dependent apoptosis and activate the NF-kappaB pathway. Immunity. 1997;7:821–30. 9430227 10.1016/s1074-7613(00)80400-8

[32] Gon S, Gatanaga T, Sendo F. Involvement of two types of TNF receptor in TNF-alpha induced neutrophil apoptosis. Microbiol Immunol. 1996;40:463–5. 8839434 10.1111/j.1348-0421.1996.tb01095.x

[33] Itoh N, Yonehara S, Ishii A, Yonehara M, Mizushima S, Sameshima M, et al. The polypeptide encoded by the cDNA for human cell surface antigen Fas can mediate apoptosis. Cell. 1991;66:233–43. 1713127 10.1016/0092-8674(91)90614-5

[34] Schneider P, Thome M, Burns K, Bodmer JL, Hofmann K, Kataoka T, et al. TRAIL receptors 1 (DR4) and 2 (DR5) signal FADD-dependent apoptosis and activate NF-kappaB. Immunity. 1997;7:831–6. 9430228 10.1016/s1074-7613(00)80401-x

[35] Kischkel FC, Lawrence DA, Tinel A, LeBlanc H, Virmani A, Schow P, et al. Death receptor recruitment of endogenous caspase-10 and apoptosis initiation in the absence of caspase-8. J Biol Chem. 2001;276:46639–46. 11583996 10.1074/jbc.M105102200

[36] Medema JP, Scaffidi C, Kischkel FC, Shevchenko A, Mann M, Krammer PH, Peter ME. FLICE is activated by association with the CD95 death-inducing signaling complex (DISC). EMBO J. 1997;16:2794–804. 9184224 10.1093/emboj/16.10.2794 PMC1169888

[37] Vanden Berghe T, van Loo G, Saelens X, Van Gurp M, Brouckaert G, Kalai M, et al. Differential signaling to apoptotic and necrotic cell death by Fas-associated death domain protein FADD. J Biol Chem. 2004;279:7925–33. 14668343 10.1074/jbc.M307807200

[38] Huang K, Zhang J, O’Neill KL, Gurumurthy CB, Quadros RM, Tu Y, Luo X. Cleavage by caspase 8 and mitochondrial membrane association activate the BH3-only Protein Bid during TRAIL-induced Apoptosis. J Biol Chem. 2016;291:11843–51. 27053107 10.1074/jbc.M115.711051 PMC4882451

[39] Morana O, Wood W, Gregory CD. The apoptosis paradox in cancer. Int J Mol Sci. 2022;23:1328–46. 10.3390/ijms23031328 PMC8836235 35163253

[40] Kaczmarek A, Vandenabeele P, Krysko DV. Necroptosis: the release of damage-associated molecular patterns and its physiological relevance. Immunity. 2013;38:209–23. 23438821 10.1016/j.immuni.2013.02.003

[41] Liu C, Zhang K, Shen H, Yao X, Sun Q, Chen G. Necroptosis: A novel manner of cell death, associated with stroke (Review). Int J Mol Med. 2018;41:624–30. 29207014 10.3892/ijmm.2017.3279

[42] Vercammen D, Beyaert R, Denecker G, Goossens V, Van Loo G, Declercq W, et al. Inhibition of caspases increases the sensitivity of L929 cells to necrosis mediated by tumor necrosis factor. J Exp Med. 1998;187:1477–85. 9565639 10.1084/jem.187.9.1477 PMC2212268

[43] Degterev A, Huang Z, Boyce M, Li Y, Jagtap P, Mizushima N, et al. Chemical inhibitor of nonapoptotic cell death with therapeutic potential for ischemic brain injury. Nat Chem Biol. 2005;1:112–9. 16408008 10.1038/nchembio711

[44] Cho YS, Challa S, Moquin D, Genga R, Ray TD, Guildford M, Chan FK. Phosphorylation-driven assembly of the RIP1-RIP3 complex regulates programmed necrosis and virus-induced inflammation. Cell. 2009;137:1112–23. 19524513 10.1016/j.cell.2009.05.037 PMC2727676

[45] He S, Wang L, Miao L, Wang T, Du F, Zhao L, Wang X. Receptor interacting protein kinase-3 determines cellular necrotic response to TNF-alpha. Cell. 2009;137:1100–11. 19524512 10.1016/j.cell.2009.05.021

[46] Zhang DW, Shao J, Lin J, Zhang N, Lu BJ, Lin SC, et al. RIP3, an energy metabolism regulator that switches TNF-induced cell death from apoptosis to necrosis. Science. 2009;325:332–6. 19498109 10.1126/science.1172308

[47] Sun L, Wang H, Wang Z, He S, Chen S, Liao D, et al. Mixed lineage kinase domain-like protein mediates necrosis signaling downstream of RIP3 kinase. Cell. 2012;148:213–27. 22265413 10.1016/j.cell.2011.11.031

[48] Zhao J, Jitkaew S, Cai Z, Choksi S, Li Q, Luo J, Liu ZG. Mixed lineage kinase domain-like is a key receptor interacting protein 3 downstream component of TNF-induced necrosis. Proc Natl Acad Sci USA. 2012;109:5322–7. 22421439 10.1073/pnas.1200012109 PMC3325682

[49] Newton K, Manning G. Necroptosis and Inflammation. Annu Rev Biochem. 2016;85:743–63. 26865533 10.1146/annurev-biochem-060815-014830

[50] Degterev A, Hitomi J, Germscheid M, Ch’en IL, Korkina O, Teng X, et al. Identification of RIP1 kinase as a specific cellular target of necrostatins. Nat Chem Biol. 2008;4:313–21. 18408713 10.1038/nchembio.83 PMC5434866

[51] Vandenabeele P, Galluzzi L, Vanden Berghe T, Kroemer G. Molecular mechanisms of necroptosis: an ordered cellular explosion. Nat Rev Mol Cell Biol. 2010;11:700–14. 20823910 10.1038/nrm2970

[52] Li J, McQuade T, Siemer AB, Napetschnig J, Moriwaki K, Hsiao YS, et al. The RIP1/RIP3 necrosome forms a functional amyloid signaling complex required for programmed necrosis. Cell. 2012;150:339–50. 22817896 10.1016/j.cell.2012.06.019 PMC3664196

[53] Golstein P, Kroemer G. Cell death by necrosis: towards a molecular definition. Trends Biochem Sci. 2007;32:37–43. 17141506 10.1016/j.tibs.2006.11.001

[54] Orozco S, Yatim N, Werner MR, Tran H, Gunja SY, Tait SW, et al. RIPK1 both positively and negatively regulates RIPK3 oligomerization and necroptosis. Cell Death Differ. 2014;21:1511–21. 24902904 10.1038/cdd.2014.76 PMC4158689

[55] Pasparakis M, Vandenabeele P. Necroptosis and its role in inflammation. Nature. 2015;517:311–20. 25592536 10.1038/nature14191

[56] Vandenabeele P, Declercq W, Van Herreweghe F, Vanden Berghe T. The role of the kinases RIP1 and RIP3 in TNF-induced necrosis. Sci Signal. 2010;3:re4. 20354226 10.1126/scisignal.3115re4

[57] Wu XN, Yang ZH, Wang XK, Zhang Y, Wan H, Song Y, et al. Distinct roles of RIP1-RIP3 hetero- and RIP3-RIP3 homo-interaction in mediating necroptosis. Cell Death Differ. 2014;21:1709–20. 24902902 10.1038/cdd.2014.77 PMC4211369

[58] Cai Z, Jitkaew S, Zhao J, Chiang HC, Choksi S, Liu J, et al. Plasma membrane translocation of trimerized MLKL protein is required for TNF-induced necroptosis. Nat Cell Biol. 2014;16:55–65. 24316671 10.1038/ncb2883 PMC8369836

[59] Chen X, Li W, Ren J, Huang D, He WT, Song Y, et al. Translocation of mixed lineage kinase domain-like protein to plasma membrane leads to necrotic cell death. Cell Res. 2014;24:105–21. 24366341 10.1038/cr.2013.171 PMC3879712

[60] Khan N, Lawlor KE, Murphy JM, Vince JE. More to life than death: molecular determinants of necroptotic and non-necroptotic RIP3 kinase signaling. Curr Opin Immunol. 2014;26:76–89. 24556404 10.1016/j.coi.2013.10.017

[61] Biswas SK, Mantovani A. Macrophage plasticity and interaction with lymphocyte subsets: cancer as a paradigm. Nat Immunol. 2010;11:889–96. 20856220 10.1038/ni.1937

[62] Steinman RM. Dendritic cells: understanding immunogenicity. Eur J Immunol. 2007;37 Supp 1:S53–60. 17972346 10.1002/eji.200737400

[63] Yatim N, Jusforgues-Saklani H, Orozco S, Schulz O, Barreira da Silva R, Reis e Sousa C, et al. RIPK1 and NF-kappaB signaling in dying cells determines cross-priming of CD8(+) T cells. Science. 2015;350:328–34. 26405229 10.1126/science.aad0395 PMC4651449

[64] Aaes TL, Kaczmarek A, Delvaeye T, De Craene B, De Koker S, Heyndrickx L, et al. Vaccination with Necroptotic Cancer Cells Induces Efficient Anti-tumor Immunity. Cell Rep. 2016;15:274–87. 27050509 10.1016/j.celrep.2016.03.037

[65] Seifert L, Werba G, Tiwari S, Giao Ly NN, Alothman S, Alqunaibit D, et al. The necrosome promotes pancreatic oncogenesis via CXCL1 and Mincle-induced immune suppression. Nature. 2016;532:245–9. 27049944 10.1038/nature17403 PMC4833566

[66] Chen Y, Smith MR, Thirumalai K, Zychlinsky A. A bacterial invasin induces macrophage apoptosis by binding directly to ICE. EMBO J. 1996;15:3853–60. 8670890 PMC452076

[67] Zychlinsky A, Prevost MC, Sansonetti PJ. Shigella flexneri induces apoptosis in infected macrophages. Nature. 1992;358:167–9. 1614548 10.1038/358167a0

[68] Brennan MA, Cookson BT. Salmonella induces macrophage death by caspase-1-dependent necrosis. Mol Microbiol. 2000;38:31–40. 11029688 10.1046/j.1365-2958.2000.02103.x

[69] Cookson BT, Brennan MA. Pro-inflammatory programmed cell death. Trends Microbiol. 2001;9:113–4. 11303500 10.1016/s0966-842x(00)01936-3

[70] He WT, Wan H, Hu L, Chen P, Wang X, Huang Z, et al. Gasdermin D is an executor of pyroptosis and required for interleukin-1beta secretion. Cell Res. 2015;25:1285–98. 26611636 10.1038/cr.2015.139 PMC4670995

[71] Shi J, Zhao Y, Wang K, Shi X, Wang Y, Huang H, et al. Cleavage of GSDMD by inflammatory caspases determines pyroptotic cell death. Nature. 2015;526:660–5. 26375003 10.1038/nature15514

[72] Li P, Allen H, Banerjee S, Franklin S, Herzog L, Johnston C, et al. Mice deficient in IL-1 beta-converting enzyme are defective in production of mature IL-1 beta and resistant to endotoxic shock. Cell. 1995;80:401–11. 7859282 10.1016/0092-8674(95)90490-5

[73] Lamkanfi M, Dixit VM. Mechanisms and functions of inflammasomes. Cell. 2014;157:1013–22. 24855941 10.1016/j.cell.2014.04.007

[74] Liston A, Masters SL. Homeostasis-altering molecular processes as mechanisms of inflammasome activation. Nat Rev Immunol. 2017;17:208–14. 28163301 10.1038/nri.2016.151

[75] Martinon F, Burns K, Tschopp J. The inflammasome: a molecular platform triggering activation of inflammatory caspases and processing of proIL-beta. Mol Cell. 2002;10:417–26. 12191486 10.1016/s1097-2765(02)00599-3

[76] Sollberger G, Strittmatter GE, Garstkiewicz M, Sand J, Beer HD. Caspase-1: the inflammasome and beyond. Innate Immun. 2014;20:115–25. 23676582 10.1177/1753425913484374

[77] Strowig T, Henao-Mejia J, Elinav E, Flavell R. Inflammasomes in health and disease. Nature. 2012;481:278–86. 22258606 10.1038/nature10759

[78] Liu X, Zhang Z, Ruan J, Pan Y, Magupalli VG, Wu H, Lieberman J. Inflammasome-activated gasdermin D causes pyroptosis by forming membrane pores. Nature. 2016;535:153–8. 27383986 10.1038/nature18629 PMC5539988

[79] Man SM, Karki R, Kanneganti TD. Molecular mechanisms and functions of pyroptosis, inflammatory caspases and inflammasomes in infectious diseases. Immunol Rev. 2017;277:61–75. 28462526 10.1111/imr.12534 PMC5416822

[80] Agard NJ, Maltby D, Wells JA. Inflammatory stimuli regulate caspase substrate profiles. Mol Cell Proteom. 2010;9:880–93. 10.1074/mcp.M900528-MCP200 PMC2871421 20173201

[81] Kayagaki N, Stowe IB, Lee BL, O’Rourke K, Anderson K, Warming S, et al. Caspase-11 cleaves gasdermin D for non-canonical inflammasome signalling. Nature. 2015;526:666–71. 26375259 10.1038/nature15541

[82] Baker PJ, Boucher D, Bierschenk D, Tebartz C, Whitney PG, D’Silva DB, et al. NLRP3 inflammasome activation downstream of cytoplasmic LPS recognition by both caspase-4 and caspase-5. Eur J Immunol. 2015;45:2918–26. 26173988 10.1002/eji.201545655

[83] Ramirez MLG, Poreba M, Snipas SJ, Groborz K, Drag M, Salvesen GS. Extensive peptide and natural protein substrate screens reveal that mouse caspase-11 has much narrower substrate specificity than caspase-1. J Biol Chem. 2018;293:7058–67. 29414788 10.1074/jbc.RA117.001329 PMC5936834

[84] Ruhl S, Broz P. Caspase-11 activates a canonical NLRP3 inflammasome by promoting K(+) efflux. Eur J Immunol. 2015;45:2927–36. 26173909 10.1002/eji.201545772

[85] Fang Y, Tian S, Pan Y, Li W, Wang Q, Tang Y, et al. Pyroptosis: a new frontier in cancer. Biomed Pharmacother. 2020;121:109595. 31710896 10.1016/j.biopha.2019.109595

[86] de Beeck KO, Van Laer L, Van Camp G. DFNA5, a gene involved in hearing loss and cancer: a review. Ann Otol Rhinol Laryngol. 2012;121:197–207. 22530481 10.1177/000348941212100310

[87] Hergueta-Redondo M, Sarrio D, Molina-Crespo A, Megias D, Mota A, Rojo-Sebastian A, et al. Gasdermin-B promotes invasion and metastasis in breast cancer cells. PLoS One. 2014;9:e90099. 24675552 10.1371/journal.pone.0090099 PMC3967990

[88] Hou J, Zhao R, Xia W, Chang CW, You Y, Hsu JM, et al. PD-L1-mediated gasdermin C expression switches apoptosis to pyroptosis in cancer cells and facilitates tumour necrosis. Nat Cell Biol. 2020;22:1264–75. 32929201 10.1038/s41556-020-0575-z PMC7653546

[89] Bagheri V, Memar B, Momtazi AA, Sahebkar A, Gholamin M, Abbaszadegan MR. Cytokine networks and their association with Helicobacter pylori infection in gastric carcinoma. J Cell Physiol. 2018;233:2791–803. 28121015 10.1002/jcp.25822

[90] Lamb A, Chen LF. Role of the Helicobacter pylori-induced inflammatory response in the development of gastric cancer. J Cell Biochem. 2013;114:491–7. 22961880 10.1002/jcb.24389 PMC3909030

[91] Li S, Liang X, Ma L, Shen L, Li T, Zheng L, et al. MiR-22 sustains NLRP3 expression and attenuates H. pylori-induced gastric carcinogenesis. Oncogene. 2018;37:884–96. 29059152 10.1038/onc.2017.381

[92] Wang Q, Wang Y, Ding J, Wang C, Zhou X, Gao W, et al. A bioorthogonal system reveals antitumour immune function of pyroptosis. Nature. 2020;579:421–6. 32188939 10.1038/s41586-020-2079-1

[93] Zhang Z, Zhang Y, Xia S, Kong Q, Li S, Liu X, et al. Gasdermin E suppresses tumour growth by activating anti-tumour immunity. Nature. 2020;579:415–20. 32188940 10.1038/s41586-020-2071-9 PMC7123794

[94] Wang Q, Imamura R, Motani K, Kushiyama H, Nagata S, Suda T. Pyroptotic cells externalize eat-me and release find-me signals and are efficiently engulfed by macrophages. Int Immunol. 2013;25:363–72. 23446850 10.1093/intimm/dxs161

[95] Ghiringhelli F, Apetoh L, Tesniere A, Aymeric L, Ma Y, Ortiz C, et al. Activation of the NLRP3 inflammasome in dendritic cells induces IL-1beta-dependent adaptive immunity against tumors. Nat Med. 2009;15:1170–8. 19767732 10.1038/nm.2028

[96] Stockwell BR, Friedmann Angeli JP, Bayir H, Bush AI, Conrad M, Dixon SJ, et al. Ferroptosis: a regulated cell death nexus linking metabolism, redox biology, and disease. Cell. 2017;171:273–85. 28985560 10.1016/j.cell.2017.09.021 PMC5685180

[97] Dolma S, Lessnick SL, Hahn WC, Stockwell BR. Identification of genotype-selective antitumor agents using synthetic lethal chemical screening in engineered human tumor cells. Cancer Cell. 2003;3:285–96. 12676586 10.1016/s1535-6108(03)00050-3

[98] Yagoda N, von Rechenberg M, Zaganjor E, Bauer AJ, Yang WS, Fridman DJ, et al. RAS-RAF-MEK-dependent oxidative cell death involving voltage-dependent anion channels. Nature. 2007;447:864–8. 17568748 10.1038/nature05859 PMC3047570

[99] Yang WS, Stockwell BR. Synthetic lethal screening identifies compounds activating iron-dependent, nonapoptotic cell death in oncogenic-RAS-harboring cancer cells. Chem Biol. 2008;15:234–45. 18355723 10.1016/j.chembiol.2008.02.010 PMC2683762

[100] Dixon SJ, Lemberg KM, Lamprecht MR, Skouta R, Zaitsev EM, Gleason CE, et al. Ferroptosis: an iron-dependent form of nonapoptotic cell death. Cell. 2012;149:1060–72. 22632970 10.1016/j.cell.2012.03.042 PMC3367386

[101] Jiang L, Hickman JH, Wang SJ, Gu W. Dynamic roles of p53-mediated metabolic activities in ROS-induced stress responses. Cell Cycle. 2015;14:2881–5. 26218928 10.1080/15384101.2015.1068479 PMC4825584

[102] Jiang L, Kon N, Li T, Wang SJ, Su T, Hibshoosh H, et al. Ferroptosis as a p53-mediated activity during tumour suppression. Nature. 2015;520:57–62. 25799988 10.1038/nature14344 PMC4455927

[103] Yang WS, SriRamaratnam R, Welsch ME, Shimada K, Skouta R, Viswanathan VS, et al. Regulation of ferroptotic cancer cell death by GPX4. Cell. 2014;156:317–31. 24439385 10.1016/j.cell.2013.12.010 PMC4076414

[104] Wang H, Cheng Y, Mao C, Liu S, Xiao D, Huang J, Tao Y. Emerging mechanisms and targeted therapy of ferroptosis in cancer. Mol Ther. 2021;29:2185–208. 33794363 10.1016/j.ymthe.2021.03.022 PMC8261167

[105] Ou Y, Wang SJ, Li D, Chu B, Gu W. Activation of SAT1 engages polyamine metabolism with p53-mediated ferroptotic responses. Proc Natl Acad Sci USA. 2016;113:E6806–E12. 27698118 10.1073/pnas.1607152113 PMC5098629

[106] Doll S, Proneth B, Tyurina YY, Panzilius E, Kobayashi S, Ingold I, et al. ACSL4 dictates ferroptosis sensitivity by shaping cellular lipid composition. Nat Chem Biol. 2017;13:91–8. 27842070 10.1038/nchembio.2239 PMC5610546

[107] Kagan VE, Mao G, Qu F, Angeli JP, Doll S, Croix CS, et al. Oxidized arachidonic and adrenic PEs navigate cells to ferroptosis. Nat Chem Biol. 2017;13:81–90. 27842066 10.1038/nchembio.2238 PMC5506843

[108] Dixon SJ, Winter GE, Musavi LS, Lee ED, Snijder B, Rebsamen M, et al. Human haploid cell genetics reveals roles for lipid metabolism genes in nonapoptotic cell death. ACS Chem Biol. 2015;10:1604–9. 25965523 10.1021/acschembio.5b00245 PMC4509420

[109] Hassannia B, Vandenabeele P, Vanden Berghe T. Targeting ferroptosis to iron out cancer. Cancer Cell. 2019;35:830–49. 31105042 10.1016/j.ccell.2019.04.002

[110] Mou Y, Wang J, Wu J, He D, Zhang C, Duan C, Li B. Ferroptosis, a new form of cell death: opportunities and challenges in cancer. J Hematol Oncol. 2019;12:34. 30925886 10.1186/s13045-019-0720-y PMC6441206

[111] Xu T, Ding W, Ji X, Ao X, Liu Y, Yu W, Wang J. Molecular mechanisms of ferroptosis and its role in cancer therapy. J Cell Mol Med. 2019;23:4900–12. 31232522 10.1111/jcmm.14511 PMC6653007

[112] Wang W, Green M, Choi JE, Gijon M, Kennedy PD, Johnson JK, et al. CD8(+) T cells regulate tumour ferroptosis during cancer immunotherapy. Nature. 2019;569:270–4. 31043744 10.1038/s41586-019-1170-y PMC6533917

[113] Joyce JA, Fearon DT. T cell exclusion, immune privilege, and the tumor microenvironment. Science. 2015;348:74–80. 25838376 10.1126/science.aaa6204

[114] Mohamed E, Al-Khami AA, Rodriguez PC. The cellular metabolic landscape in the tumor milieu regulates the activity of myeloid infiltrates. Cell Mol Immunol. 2018;15:421–7. 29568118 10.1038/s41423-018-0001-7 PMC6068094

[115] Thommen DS, Schumacher TN. T cell dysfunction in cancer. Cancer Cell. 2018;33:547–62. 29634943 10.1016/j.ccell.2018.03.012 PMC7116508

[116] Pepino MY, Kuda O, Samovski D, Abumrad NA. Structure-function of CD36 and importance of fatty acid signal transduction in fat metabolism. Annu Rev Nutr. 2014;34:281–303. 24850384 10.1146/annurev-nutr-071812-161220 PMC4329921

[117] Silverstein RL, Febbraio M. CD36, a scavenger receptor involved in immunity, metabolism, angiogenesis, and behavior. Sci Signal. 2009;2:re3. 19471024 10.1126/scisignal.272re3 PMC2811062

[118] Festa RA, Thiele DJ. Copper: an essential metal in biology. Curr Biol. 2011;21:R877–83. 22075424 10.1016/j.cub.2011.09.040 PMC3718004

[119] Xue Q, Kang R, Klionsky DJ, Tang D, Liu J, Chen X. Copper metabolism in cell death and autophagy. Autophagy. 2023;19:2175–95. 37055935 10.1080/15548627.2023.2200554 PMC10351475

[120] Nagai M, Vo NH, Shin Ogawa L, Chimmanamada D, Inoue T, Chu J, et al. The oncology drug elesclomol selectively transports copper to the mitochondria to induce oxidative stress in cancer cells. Free Radic Biol Med. 2012;52:2142–50. 22542443 10.1016/j.freeradbiomed.2012.03.017

[121] Tsvetkov P, Detappe A, Cai K, Keys HR, Brune Z, Ying W, et al. Mitochondrial metabolism promotes adaptation to proteotoxic stress. Nat Chem Biol. 2019;15:681–9. 31133756 10.1038/s41589-019-0291-9 PMC8183600

[122] Tsvetkov P, Coy S, Petrova B, Dreishpoon M, Verma A, Abdusamad M, et al. Copper induces cell death by targeting lipoylated TCA cycle proteins. Science. 2022;375:1254–61. 35298263 10.1126/science.abf0529 PMC9273333

[123] Yaman M, Kaya G, Yekeler H. Distribution of trace metal concentrations in paired cancerous and non-cancerous human stomach tissues. World J Gastroenterol. 2007;13:612–8. 17278230 10.3748/wjg.v13.i4.612 PMC4065986

[124] Basu S, Singh MK, Singh TB, Bhartiya SK, Singh SP, Shukla VK. Heavy and trace metals in carcinoma of the gallbladder. World J Surg. 2013;37:2641–6. 23942528 10.1007/s00268-013-2164-9

[125] Wang W, Wang X, Luo J, Chen X, Ma K, He H, et al. Serum copper level and the copper-to-zinc ratio could be useful in the prediction of lung cancer and its prognosis: a case-control study in Northeast China. Nutr Cancer. 2021;73:1908–15. 32896161 10.1080/01635581.2020.1817957

[126] Kosova F, Cetin B, Akinci M, Aslan S, Seki A, Pirhan Y, Ari Z. Serum copper levels in benign and malignant thyroid diseases. Bratisl Lek Listy. 2012;113:718–20. 23173630 10.4149/bll_2012_162

[127] Pavithra V, Sathisha TG, Kasturi K, Mallika DS, Amos SJ, Ragunatha S. Serum levels of metal ions in female patients with breast cancer. J Clin Diagn Res. 2015;9:BC25–c7. 25737978 10.7860/JCDR/2015/11627.5476 PMC4347069

[128] Saleh SAK, Adly HM, Abdelkhaliq AA, Nassir AM. Serum levels of selenium, zinc, copper, manganese, and iron in prostate cancer patients. Curr Urol. 2020;14:44–9. 32398996 10.1159/000499261 PMC7206590

[129] Towers CG, Wodetzki D, Thorburn A. Autophagy and cancer: modulation of cell death pathways and cancer cell adaptations. J Cell Biol. 2020;219:e201909033. 10.1083/jcb.201909033 PMC7039213 31753861

[130] Peng F, Liao M, Qin R, Zhu S, Peng C, Fu L, et al. Regulated cell death (RCD) in cancer: key pathways and targeted therapies. Signal Transduct Target Ther. 2022;7:286. 35963853 10.1038/s41392-022-01110-y PMC9376115

[131] Erazo T, Lorente M, Lopez-Plana A, Munoz-Guardiola P, Fernandez-Nogueira P, Garcia-Martinez JA, et al. The new antitumor drug ABTL0812 inhibits the Akt/mTORC1 axis by upregulating tribbles-3 pseudokinase. Clin Cancer Res. 2016;22:2508–19. 26671995 10.1158/1078-0432.CCR-15-1808

[132] Wang J, Liang D, Zhang XP, He CF, Cao L, Zhang SQ, et al. Novel PI3K/Akt/mTOR signaling inhibitor, W922, prevents colorectal cancer growth via the regulation of autophagy. Int J Oncol. 2021;58:70–82. 33367926 10.3892/ijo.2020.5151 PMC7721087

[133] Siqueira EDS, Concato VM, Tomiotto-Pellissier F, Silva TF, Bortoleti B, Goncalves MD, et al. Trans-chalcone induces death by autophagy mediated by p53 up-regulation and beta-catenin down-regulation on human hepatocellular carcinoma HuH7.5 cell line. Phytomedicine. 2021;80:153373. 33096451 10.1016/j.phymed.2020.153373

[134] Cha YE, Park R, Jang M, Park YI, Yamamoto A, Oh WK, et al. 6-azauridine induces autophagy-mediated cell death via a p53- and AMPK-dependent pathway. Int J Mol Sci. 2021;22:2947–56. 10.3390/ijms22062947 PMC8000275 33799444

[135] Wang N, Wang H, Li L, Li Y, Zhang R. beta-asarone inhibits amyloid-beta by promoting autophagy in a cell model of Alzheimer’s disease. Front Pharm. 2019;10:1529. 10.3389/fphar.2019.01529 PMC6979317 32009952

[136] Huang PJ, Chiu CC, Hsiao MH, Yow JL, Tzang BS, Hsu TC. Potential of antiviral drug oseltamivir for the treatment of liver cancer. Int J Oncol. 2021;59:109–28. 10.3892/ijo.2021.5289 PMC8651232 34859259

[137] Dai Y, Lawrence TS, Xu L. Overcoming cancer therapy resistance by targeting inhibitors of apoptosis proteins and nuclear factor-kappa B. Am J Transl Res. 2009;1:1–15. 19966933 PMC2776288

[138] Tron AE, Belmonte MA, Adam A, Aquila BM, Boise LH, Chiarparin E, et al. Discovery of Mcl-1-specific inhibitor AZD5991 and preclinical activity in multiple myeloma and acute myeloid leukemia. Nat Commun. 2018;9:5341. 30559424 10.1038/s41467-018-07551-w PMC6297231

[139] Peterson TJ, Orozco J, Buege M. Selinexor: a first-in-class nuclear export inhibitor for management of multiply relapsed multiple myeloma. Ann Pharmacother. 2020;54:577–82. 31793336 10.1177/1060028019892643 PMC8498942

[140] Abdul Razak AR, Mau-Soerensen M, Gabrail NY, Gerecitano JF, Shields AF, Unger TJ, et al. First-in-class, first-in-human phase I study of selinexor, a selective inhibitor of nuclear export, in patients with advanced solid tumors. J Clin Oncol. 2016;34:4142–50. 26926685 10.1200/JCO.2015.65.3949 PMC5562433

[141] Shishodia G, Koul S, Dong Q, Koul HK. Tetrandrine (TET) induces death receptors Apo trail R1 (DR4) and Apo trail R2 (DR5) and sensitizes prostate cancer cells to TRAIL-induced apoptosis. Mol Cancer Ther. 2018;17:1217–28. 29549167 10.1158/1535-7163.MCT-17-1157 PMC10186773

[142] Ralff MD, Lulla AR, Wagner J, El-Deiry WS. ONC201: a new treatment option being tested clinically for recurrent glioblastoma. Transl Cancer Res. 2017;6:S1239–S43. 30175049 10.21037/tcr.2017.10.03 PMC6117120

[143] Sp N, Kang DY, Jo ES, Rugamba A, Kim WS, Park YM, et al. Tannic acid promotes TRAIL-induced extrinsic apoptosis by regulating mitochondrial ROS in human embryonic carcinoma cells. Cells. 2020;9:282–98. 10.3390/cells9020282 PMC7072125 31979292

[144] Lee YJ, Nam HS, Cho MK, Lee SH. Arctigenin induces necroptosis through mitochondrial dysfunction with CCN1 upregulation in prostate cancer cells under lactic acidosis. Mol Cell Biochem. 2020;467:45–56. 32065351 10.1007/s11010-020-03699-6

[145] Lu Z, Wu C, Zhu M, Song W, Wang H, Wang J, et al. Ophiopogonin D’ induces RIPK1‑dependent necroptosis in androgen‑dependent LNCaP prostate cancer cells. Int J Oncol. 2020;56:439–47. 31894265 10.3892/ijo.2019.4945 PMC6959467

[146] Tong X, Tang R, Xiao M, Xu J, Wang W, Zhang B, et al. Targeting cell death pathways for cancer therapy: recent developments in necroptosis, pyroptosis, ferroptosis, and cuproptosis research. J Hematol Oncol. 2022;15:174. 36482419 10.1186/s13045-022-01392-3 PMC9733270

[147] Gong Y, Fan Z, Luo G, Yang C, Huang Q, Fan K, et al. The role of necroptosis in cancer biology and therapy. Mol Cancer. 2019;18:100. 31122251 10.1186/s12943-019-1029-8 PMC6532150

[148] Meng MB, Wang HH, Cui YL, Wu ZQ, Shi YY, Zaorsky NG, et al. Necroptosis in tumorigenesis, activation of anti-tumor immunity, and cancer therapy. Oncotarget. 2016;7:57391–413. 27429198 10.18632/oncotarget.10548 PMC5302997

[149] Fulda S. Therapeutic exploitation of necroptosis for cancer therapy. Semin Cell Dev Biol. 2014;35:51–6. 25065969 10.1016/j.semcdb.2014.07.002

[150] Zhang Z, Zhang Z, Li Q, Jiao H, Chong D, Sun X, et al. Shikonin induces necroptosis by reactive oxygen species activation in nasopharyngeal carcinoma cell line CNE-2Z. J Bioenerg Biomembr. 2017;49:265–72. 28547157 10.1007/s10863-017-9714-z

[151] Loveless R, Bloomquist R, Teng Y. Pyroptosis at the forefront of anticancer immunity. J Exp Clin Cancer Res. 2021;40:264. 34429144 10.1186/s13046-021-02065-8 PMC8383365

[152] Hua Y, Zheng Y, Yao Y, Jia R, Ge S, Zhuang A. Metformin and cancer hallmarks: shedding new lights on therapeutic repurposing. J Transl Med. 2023;21:403. 37344841 10.1186/s12967-023-04263-8 PMC10286395

[153] Draganov D, Gopalakrishna-Pillai S, Chen YR, Zuckerman N, Moeller S, Wang C, et al. Modulation of P2X4/P2X7/Pannexin-1 sensitivity to extracellular ATP via Ivermectin induces a non-apoptotic and inflammatory form of cancer cell death. Sci Rep. 2015;5:16222. 26552848 10.1038/srep16222 PMC4639773

[154] Tan YF, Wang M, Chen ZY, Wang L, Liu XH. Inhibition of BRD4 prevents proliferation and epithelial-mesenchymal transition in renal cell carcinoma via NLRP3 inflammasome-induced pyroptosis. Cell Death Dis. 2020;11:239. 32303673 10.1038/s41419-020-2431-2 PMC7165180

[155] Qiao L, Wu X, Zhang J, Liu L, Sui X, Zhang R, et al. alpha-NETA induces pyroptosis of epithelial ovarian cancer cells through the GSDMD/caspase-4 pathway. FASEB J. 2019;33:12760–7. 31480859 10.1096/fj.201900483RR

[156] Zhou W, Zhao L, Wang H, Liu X, Liu Y, Xu K, et al. Pyroptosis: a promising target for lung cancer therapy. Chin Med J Pulm Crit Care Med. 2023;1:94–101. 39170826 10.1016/j.pccm.2023.03.001 PMC11332860

[157] Li Y, Wang W, Li A, Huang W, Chen S, Han F, Wang L. Dihydroartemisinin induces pyroptosis by promoting the AIM2/caspase-3/DFNA5 axis in breast cancer cells. Chem Biol Interact. 2021;340:109434. 33689708 10.1016/j.cbi.2021.109434

[158] Su Y, Zhao B, Zhou L, Zhang Z, Shen Y, Lv H, et al. Ferroptosis, a novel pharmacological mechanism of anti-cancer drugs. Cancer Lett. 2020;483:127–36. 32067993 10.1016/j.canlet.2020.02.015

[159] Shibata Y, Yasui H, Higashikawa K, Miyamoto N, Kuge Y. Erastin, a ferroptosis-inducing agent, sensitized cancer cells to X-ray irradiation via glutathione starvation in vitro and in vivo. PLoS One. 2019;14:e0225931. 31800616 10.1371/journal.pone.0225931 PMC6892486

[160] Lu B, Chen XB, Ying MD, He QJ, Cao J, Yang B. The role of ferroptosis in cancer development and treatment response. Front Pharm. 2017;8:992. 10.3389/fphar.2017.00992 PMC5770584 29375387

[161] Dixon SJ, Patel DN, Welsch M, Skouta R, Lee ED, Hayano M, et al. Pharmacological inhibition of cystine-glutamate exchange induces endoplasmic reticulum stress and ferroptosis. Elife 2014;3:e02523. 24844246 10.7554/eLife.02523 PMC4054777

[162] Nie Q, Hu Y, Yu X, Li X, Fang X. Induction and application of ferroptosis in cancer therapy. Cancer Cell Int. 2022;22:12–30. 10.1186/s12935-021-02366-0 PMC8742449 34996454

[163] Veglia Tranchese R, Battista S, Cerchia L, Fedele M. Ferroptosis in cancer: epigenetic control and therapeutic opportunities. Biomolecules. 2024;14. 10.3390/biom14111443 PMC11592303 39595619

[164] Liu WQ, Lin WR, Yan L, Xu WH, Yang J. Copper homeostasis and cuproptosis in cancer immunity and therapy. Immunol Rev. 2024;321:211–27. 37715546 10.1111/imr.13276

[165] Cen D, Brayton D, Shahandeh B, Meyskens FL Jr., Farmer PJ. Disulfiram facilitates intracellular Cu uptake and induces apoptosis in human melanoma cells. J Med Chem. 2004;47:6914–20. 15615540 10.1021/jm049568z

[166] Zhao Y, Zhao B, Zhu S. Disulfiram/copper activates ER stress to promote immunogenic cell death of oral squamous cell carcinoma. Cell Biochem Biophys. 2024;82:1291–8. 38727783 10.1007/s12013-024-01283-z

[167] Wang Y, Chen Y, Zhang J, Yang Y, Fleishman JS, Wang Y, et al. Cuproptosis: a novel therapeutic target for overcoming cancer drug resistance. Drug Resist Updat. 2024;72:101018. 37979442 10.1016/j.drup.2023.101018

[168] Yang Y, Liang S, Geng H, Xiong M, Li M, Su Q, et al. Proteomics revealed the crosstalk between copper stress and cuproptosis, and explored the feasibility of curcumin as anticancer copper ionophore. Free Radic Biol Med. 2022;193:638–47. 36395954 10.1016/j.freeradbiomed.2022.11.023

[169] Feng Y, Yang Z, Wang J, Zhao H. Cuproptosis: unveiling a new frontier in cancer biology and therapeutics. Cell Commun Signal. 2024;22:249. 38693584 10.1186/s12964-024-01625-7 PMC11064406

[170] Tang D, Kroemer G, Kang R. Targeting cuproplasia and cuproptosis in cancer. Nat Rev Clin Oncol. 2024;21:370–88. 38486054 10.1038/s41571-024-00876-0

[171] Yang W, Wang Y, Huang Y, Yu J, Wang T, Li C, et al. 4-Octyl itaconate inhibits aerobic glycolysis by targeting GAPDH to promote cuproptosis in colorectal cancer. Biomed Pharmacother. 2023;159:114301. 36706634 10.1016/j.biopha.2023.114301

[172] Lee SY, Seo JH, Kim S, Hwang C, Jeong DI, Park J, et al. Cuproptosis-inducible chemotherapeutic/cascade catalytic reactor system for combating with breast cancer. Small. 2023;19:e2301402. 37162448 10.1002/smll.202301402

[173] Guo B, Yang F, Zhang L, Zhao Q, Wang W, Yin L, et al. Cuproptosis induced by ROS responsive nanoparticles with elesclomol and copper combined with alphaPD-L1 for enhanced cancer immunotherapy. Adv Mater. 2023;35:e2212267. 36916030 10.1002/adma.202212267

[174] Noh D, Lee H, Lee S, Sun IC, Yoon HY. Copper-based nanomedicines for cuproptosis-mediated effective cancer treatment. Biomater Res. 2024;28:0094. 39430913 10.34133/bmr.0094 PMC11486892

[175] Merlo LM, Pepper JW, Reid BJ, Maley CC. Cancer as an evolutionary and ecological process. Nat Rev Cancer. 2006;6:924–35. 17109012 10.1038/nrc2013

[176] Jahanban-Esfahlan R, Seidi K, Monhemi H, Adli ADF, Minofar B, Zare P, et al. RGD delivery of truncated coagulase to tumor vasculature affords local thrombotic activity to induce infarction of tumors in mice. Sci Rep. 2017;7:8126. 28811469 10.1038/s41598-017-05326-9 PMC5557930

[177] Rodriguez-Ruiz ME, Vitale I, Harrington KJ, Melero I, Galluzzi L. Immunological impact of cell death signaling driven by radiation on the tumor microenvironment. Nat Immunol. 2020;21:120–34. 31873291 10.1038/s41590-019-0561-4

[178] Gadiyar V, Lahey KC, Calianese D, Devoe C, Mehta D, Bono K, et al. Cell death in the tumor microenvironment: implications for cancer immunotherapy. Cells. 2020;9:2207–30. 10.3390/cells9102207 PMC7599747 33003477

[179] Liu J, Hong M, Li Y, Chen D, Wu Y, Hu Y. Programmed cell death tunes tumor immunity. Front Immunol. 2022;13:847345. 35432318 10.3389/fimmu.2022.847345 PMC9005769

[180] Niu X, Chen L, Li Y, Hu Z, He F. Ferroptosis, necroptosis, and pyroptosis in the tumor microenvironment: Perspectives for immunotherapy of SCLC. Semin Cancer Biol. 2022;86:273–85. 35288298 10.1016/j.semcancer.2022.03.009

[181] Zhang Z, Zhang F, Xie W, Niu Y, Wang H, Li G, et al. Induced necroptosis and its role in cancer immunotherapy. Int J Mol Sci. 2024;25:10760–74. 10.3390/ijms251910760 PMC11477008 39409087

[182] Du T, Gao J, Li P, Wang Y, Qi Q, Liu X, et al. Pyroptosis, metabolism, and tumor immune microenvironment. Clin Transl Med. 2021;11:e492. 34459122 10.1002/ctm2.492 PMC8329701

[183] Tan G, Huang C, Chen J, Zhi F. HMGB1 released from GSDME-mediated pyroptotic epithelial cells participates in the tumorigenesis of colitis-associated colorectal cancer through the ERK1/2 pathway. J Hematol Oncol. 2020;13:149. 33160389 10.1186/s13045-020-00985-0 PMC7648939

[184] Wang S, Chang CW, Huang J, Zeng S, Zhang X, Hung MC, Hou J. Gasdermin C sensitizes tumor cells to PARP inhibitor therapy in cancer models. J Clin Invest. 2024;134:e166841. 10.1172/JCI166841 PMC10760963 37883181

[185] Erkes DA, Cai W, Sanchez IM, Purwin TJ, Rogers C, Field CO, et al. Mutant BRAF and MEK Inhibitors Regulate the Tumor Immune Microenvironment via Pyroptosis. Cancer Discov. 2020;10:254–69. 31796433 10.1158/2159-8290.CD-19-0672 PMC7007378

[186] Hou J, Li T, Hsu JM, Zhang X, Hung MC. Gasdermins and cancers. Semin Immunol. 2023;70:101833. 37647772 10.1016/j.smim.2023.101833

[187] Efimova I, Catanzaro E, Van der Meeren L, Turubanova VD, Hammad H, Mishchenko TA, et al. Vaccination with early ferroptotic cancer cells induces efficient antitumor immunity. J Immunother Cancer. 2020;8:e001369. 10.1136/jitc-2020-001369 PMC7668384 33188036

[188] Ramakrishnan R, Tyurin VA, Veglia F, Condamine T, Amoscato A, Mohammadyani D, et al. Oxidized lipids block antigen cross-presentation by dendritic cells in cancer. J Immunol. 2014;192:2920–31. 24554775 10.4049/jimmunol.1302801 PMC3998104

[189] Friedmann Angeli JP, Krysko DV, Conrad M. Ferroptosis at the crossroads of cancer-acquired drug resistance and immune evasion. Nat Rev Cancer. 2019;19:405–14. 31101865 10.1038/s41568-019-0149-1

[190] Yang Y, Wang Y, Guo L, Gao W, Tang TL, Yan M. Interaction between macrophages and ferroptosis. Cell Death Dis. 2022;13:355. 35429990 10.1038/s41419-022-04775-z PMC9013379

[191] Shen Z, Song J, Yung BC, Zhou Z, Wu A, Chen X. Emerging strategies of cancer therapy based on ferroptosis. Adv Mater. 2018;30:e1704007. 29356212 10.1002/adma.201704007 PMC6377162

[192] Cui K, Wang K, Huang Z. Ferroptosis and the tumor microenvironment. J Exp Clin Cancer Res. 2024;43:315. 39614322 10.1186/s13046-024-03235-0 PMC11607824

[193] Zhang X, Tang B, Luo J, Yang Y, Weng Q, Fang S, et al. Cuproptosis, ferroptosis and PANoptosis in tumor immune microenvironment remodeling and immunotherapy: culprits or new hope. Mol Cancer. 2024;23:255. 39543600 10.1186/s12943-024-02130-8 PMC11566504

[194] Tong T, Zhang J, Zhu X, Hui P, Wang Z, Wu Q, et al. Prognostic autophagy-related model revealed by integrating single-cell RNA sequencing data and bulk gene profiles in gastric cancer. Front Cell Dev Biol. 2021;9:729485. 35083210 10.3389/fcell.2021.729485 PMC8785981

[195] Alexander BM, Cloughesy TF. Adult glioblastoma. J Clin Oncol. 2017;35:2402–9. 28640706 10.1200/JCO.2017.73.0119

[196] Chu F, Wu P, Mu M, Hu S, Niu C. MGCG regulates glioblastoma tumorigenicity via hnRNPK/ATG2A and promotes autophagy. Cell Death Dis. 2023;14:443. 37460467 10.1038/s41419-023-05959-x PMC10352271

[197] Zhao Z, Liu H, Zhou X, Fang D, Ou X, Ye J, et al. Necroptosis-related lncRNAs: predicting prognosis and the distinction between the cold and hot tumors in gastric cancer. J Oncol. 2021;2021:6718443. 34790235 10.1155/2021/6718443 PMC8592775

[198] Wang N, Liu D. Identification and validation a necroptosis‑related prognostic signature and associated regulatory axis in stomach adenocarcinoma. Onco Targets Ther. 2021;14:5373–83. 34880629 10.2147/OTT.S342613 PMC8648279

[199] Zhao C, Xiong K, Adam A, Ji Z, Li X. Necroptosis identifies novel molecular phenotypes and influences tumor immune microenvironment of lung adenocarcinoma. Front Immunol. 2022;13:934494. 35911707 10.3389/fimmu.2022.934494 PMC9331758

[200] Ye Y, Dai Q, Qi H. A novel defined pyroptosis-related gene signature for predicting the prognosis of ovarian cancer. Cell Death Discov. 2021;7:71. 33828074 10.1038/s41420-021-00451-x PMC8026591

[201] Zhang Q, Tan Y, Zhang J, Shi Y, Qi J, Zou D, Ci W. Pyroptosis-related signature predicts prognosis and immunotherapy efficacy in muscle-invasive bladder cancer. Front Immunol. 2022;13:782982. 35479097 10.3389/fimmu.2022.782982 PMC9035667

[202] Huang G, Zhou J, Chen J, Liu G. Identification of pyroptosis related subtypes and tumor microenvironment infiltration characteristics in breast cancer. Sci Rep. 2022;12:10640. 35739182 10.1038/s41598-022-14897-1 PMC9226023

[203] Li G, Lei J, Xu D, Yu W, Bai J, Wu G. Integrative analyses of ferroptosis and immune related biomarkers and the osteosarcoma associated mechanisms. Sci Rep. 2023;13:5770. 37031292 10.1038/s41598-023-33009-1 PMC10082853

[204] Fan X, Zhong Y, Yuan F, Zhang L, Cai Y, Liao L. A ferroptosis-related prognostic model with excellent clinical performance based on the exploration of the mechanism of oral squamous cell carcinoma progression. Sci Rep. 2023;13:1461. 36702843 10.1038/s41598-023-27676-3 PMC9880000

[205] Wu Z, Lu Z, Li L, Ma M, Long F, Wu R, et al. Identification and validation of ferroptosis-related LncRNA signatures as a novel prognostic model for colon cancer. Front Immunol. 2021;12:783362. 35154072 10.3389/fimmu.2021.783362 PMC8826443

[206] Gohil SH, Iorgulescu JB, Braun DA, Keskin DB, Livak KJ. Applying high-dimensional single-cell technologies to the analysis of cancer immunotherapy. Nat Rev Clin Oncol. 2021;18:244–56. 33277626 10.1038/s41571-020-00449-x PMC8415132

[207] Salmon H, Remark R, Gnjatic S, Merad M. Host tissue determinants of tumour immunity. Nat Rev Cancer. 2019;19:215–27. 30867580 10.1038/s41568-019-0125-9 PMC7787168

[208] Zhao T, Guo Y, Li J. Identification and experimental validation of cuproptosis regulatory program in a sepsis immune microenvironment through a combination of single-cell and bulk RNA sequencing. Front Immunol. 2024;15:1336839. 38947313 10.3389/fimmu.2024.1336839 PMC11211538

[209] Yang C, Guo Y, Wu Z, Huang J, Xiang B. Comprehensive analysis of cuproptosis-related genes in prognosis and immune infiltration of hepatocellular carcinoma based on bulk and single-cell RNA sequencing data. Cancers (Basel). 2022;14:5713–30. 10.3390/cancers14225713 PMC9688556 36428805

[210] Blockhuys S, Celauro E, Hildesjo C, Feizi A, Stal O, Fierro-Gonzalez JC, Wittung-Stafshede P. Defining the human copper proteome and analysis of its expression variation in cancers. Metallomics. 2017;9:112–23. 27942658 10.1039/c6mt00202a

[211] ZHAO Xiaole QB, SHAO Quan. Expression and prognostic value of cuprotosis-related genes in liver cancer. Cancer Res Prev Treat. 2023;50:140–5.

[212] Kramer R, Cohen D. Functional genomics to new drug targets. Nat Rev Drug Discov. 2004;3:965–72. 15520818 10.1038/nrd1552

[213] Spaethling JM, Eberwine JH. Single-cell transcriptomics for drug target discovery. Curr Opin Pharm. 2013;13:786–90. 10.1016/j.coph.2013.04.011 23725882

